# Contextual Interactions in Grating Plaid Configurations Are Explained by Natural Image Statistics and Neural Modeling

**DOI:** 10.3389/fnsys.2016.00078

**Published:** 2016-10-04

**Authors:** Udo A. Ernst, Alina Schiffer, Malte Persike, Günter Meinhardt

**Affiliations:** ^1^Computational Neuroscience Lab, Department of Physics, Institute for Theoretical Physics, University of BremenBremen, Germany; ^2^Methods Section, Department of Psychology, Johannes Gutenberg University MainzMainz, Germany

**Keywords:** natural image statistics, network model, contextual interactions, visual perception, feature integration, visual cortex

## Abstract

Processing natural scenes requires the visual system to integrate local features into global object descriptions. To achieve coherent representations, the human brain uses statistical dependencies to guide weighting of local feature conjunctions. Pairwise interactions among feature detectors in early visual areas may form the early substrate of these local feature bindings. To investigate local interaction structures in visual cortex, we combined psychophysical experiments with computational modeling and natural scene analysis. We first measured contrast thresholds for 2 × 2 grating patch arrangements (plaids), which differed in spatial frequency composition (low, high, or mixed), number of grating patch co-alignments (0, 1, or 2), and inter-patch distances (1° and 2° of visual angle). Contrast thresholds for the different configurations were compared to the prediction of probability summation (PS) among detector families tuned to the four retinal positions. For 1° distance the thresholds for all configurations were larger than predicted by PS, indicating inhibitory interactions. For 2° distance, thresholds were significantly lower compared to PS when the plaids were homogeneous in spatial frequency and orientation, but not when spatial frequencies were mixed or there was at least one misalignment. Next, we constructed a neural population model with horizontal laminar structure, which reproduced the detection thresholds after adaptation of connection weights. Consistent with prior work, contextual interactions were medium-range inhibition and long-range, orientation-specific excitation. However, inclusion of orientation-specific, inhibitory interactions between populations with different spatial frequency preferences were crucial for explaining detection thresholds. Finally, for all plaid configurations we computed their likelihood of occurrence in natural images. The likelihoods turned out to be inversely related to the detection thresholds obtained at larger inter-patch distances. However, likelihoods were almost independent of inter-patch distance, implying that natural image statistics could not explain the crowding-like results at short distances. This failure of natural image statistics to resolve the patch distance modulation of plaid visibility remains a challenge to the approach.

## 1. Introduction

Visual scenes are composed of many objects which usually extend over large regions in the visual field. However, since visual information is represented in the early visual system as a collection of isolated local features, one of the most challenging tasks for our brain is to integrate this information into coherent percepts. This task is performed by a hierarchical and recurrent network, which builds increasingly complex representations of visual scenes as information propagates to downstream visual areas (Lamme and Roelfsema, 2000; Roelfsema et al., 2002). The corresponding computations are highly non-linear (Adini et al., 1997), and even the first stages of this network are still not well understood.

For more than a century, psychophysical studies have strived to identify principles of feature integration: starting from the first attempt at quantifying the laws of feature integration by the Gestalt psychologists (Metzger, 2006), a large body of facts has been assembled which describes elementary feature integration processes in the early visual system (Ehrenstein et al., 2003). Most of this work uses oriented and localized gratings like Gabor patches since these stimuli are known to drive neurons in primary visual cortex well (Hubel and Wiesel, 1962). These are typically set into context with one or more flanking patches in various spatial configurations. Prominent findings using these stimuli include threshold modulation in collinear configurations, exhibiting suppression at small element distances, and facilitation at larger element distances (Polat and Sagi, 1993). The range of these effects typically scales almost linearly in dependence on the spatial frequency of the patches (Polat and Sagi, 1994). In addition, there is also a strong dependence on stimulus contrast, with facilitation prevailing at low contrasts and suppression observed with high contrasts (Mizobe et al., 2001). In addition to these effects, element density also plays an important role. The closer single elements in a scene are to each other, the more difficult a target element, which is typically placed in the center of such an arrangement, becomes to perceive. This effect is commonly referred to as *crowding* (Whitney and Levi, 2011).

The findings on the behavioral level have been complemented by anatomical and physiological studies. In primary visual cortex, neurons are tuned to the orientation of stimuli inside a small region in the visual field, which is termed the “classical receptive field” (shorthand: cRF). Neurons with different preferred orientations between 0 and 180° are organized into orientation hypercolumns (Hubel and Wiesel, 1962); additionally, each hypercolumn separates into populations with low or high spatial frequency preference (Shmuel and Grinvald, 1996). Contextual interactions in psychophysical studies have been related to the so-called “non-classical” receptive fields (shorthand: ncRFs): modulations of neural responses by stimuli positioned outside the cRF, in addition to a stimulus inside the cRF (Haider et al., 2010; Ernst, 2013). These physiological effects turned out to be (partly) compatible with the behavioral evidence: for example, in colinear configurations, suppression and facilitation depends on grating contrast (Mizobe et al., 2001). Also, interactions between two oriented line segments at different visual field positions (Kapadia et al., 2000) resemble interaction patterns (“association fields”) proposed to explain contour integration (Field et al., 1993; Kovacs, 1996). For mediating these effects, anatomical studies identified connection structures putatively responsible for ncRFs, such as orientation-specific long-range excitatory horizontal interactions (Bosking et al., 1997) for enhancing collinear configurations, or short-range feedback projections from higher visual areas targeting inhibitory circuits (Johnson and Burkhalter, 1996; Lamme et al., 1998; Hupé et al., 2001; Callaway, 2004) for surround suppression. In addition, there is a dense and not yet fully understood network within a cortical column. In particular, any projections entering a cortical column may target inhibitory or excitatory populations, thus being able to exert a potentially positive or negative modulation.

While models constructed from anatomical and physiological knowledge were reasonably successful in explaining a range of extra-classical receptive field properties (for an overview, see Ernst, 2013), a different idea is to understand feature integration processes from first principles. This includes deriving stylized facts about ncRFs from postulating that neurons in visual cortex perform probabilistic inference on visual scenes (Lochmann et al., 2012), or from requiring visual cortex to construct a sparse representation of natural stimuli (Zhu and Rozell, 2013). It has also been shown that natural image statistics explains fundamental laws in feature integration, such as the law of good continuation by demonstrating a close match between contour statistics and the shape of the association field used by the visual system for contour integration (Geisler et al., 2001; Geisler and Perry, 2009).

Taking together these results from the past 20 years, a coherent account of feature integration begins to emerge. However, since experimental work often uses structurally simple stimuli, we still do not understand enough about how more complex stimulus configurations, or stimuli involving two or more elementary features, are represented and processed. To fill this gap, we present a study which analyzes feature integration with a combination of methods (experiment, modeling, image statistics) spanning a range of observation levels (psychophysics, neural network simulations, external world), thus aiming at a unifying perspective. In particular, we focus on the following questions: How do different feature dimensions interact, and how are they processed by the visual system? What kind of neural interactions would be required to explain the corresponding effects? What does behavior tell us about computations performed by the visual system, and are the observed effects linked to the higher-order statistics of the “typical” stimuli processed by the visual system?

For this purpose, we extended the standard experimental paradigm of using visual stimuli consisting of strings of oriented grating patches to patches arranged in more complex, two-by-two element plaids. This enables us to investigate the interplay of interactions along two orthogonal axes in visual space. We introduced spatial frequency as a second feature dimension besides orientation and first quantified human detection thresholds for different plaid configurations with varying inter-patch distances. Next we reproduced human behavior in a simplistic neural network and identified interaction structures which are capable of explaining our experimental data. Finally, we compared the statistics of the plaid configurations in natural images to human behavior and tested the hypothesis that visual stimuli occurring more frequently are detected more easily. Our results turned out to be compatible with prior work and in addition reveal three major findings going beyond well-established facts:

- Detection thresholds are perfectly explained by pairwise couplings in a structurally simple model; thus no higher-order interaction schemes are required.
- Interactions between feature detectors with different preferred spatial frequencies must be both suppressive, and orientation-specific.
- For larger inter-patch distances, detection performance is inversely related to plaid likelihood (ratio) in natural images.

By obtaining these results in a common framework encompassing experiment, modeling, and image statistics, our study directly addresses two main goals of this special issue in Frontiers, namely that “brain activity is predicted from [e.g.,] stimuli (encoding),” and that “subjective/cognitive states are predicted from brain activity (decoding).”

## 2. Materials and methods

### 2.1. Experiments

#### 2.1.1. Outline

We created 2D spatial arrangements of four grating patches (“plaids”) to study the impact of spatial distance, spatial frequency homogeneity and orientation alignment on the detectability of the patch arrangement. Two spatial frequencies and two orientations were used for the grating patches. In preparatory measurements the carrier frequencies were determined such that all four patches were equally detectable when presented individually on the spatial position grids. Contrasts thresholds were measured for plaids with 0, 1, or 2 orientation alignments in spatial frequency homogeneous and inhomogeneous configurations, and with near and far distance between patches. To compare with a benchmark, the contrast thresholds were tested against the prediction derived from probability summation among the four locations of a plaid arrangement. This test was used to indicate whether the specific parameter combination of a plaid yielded inhibition, facilitation or independent feature processing.

#### 2.1.2. Stimuli

**Figure 1:**
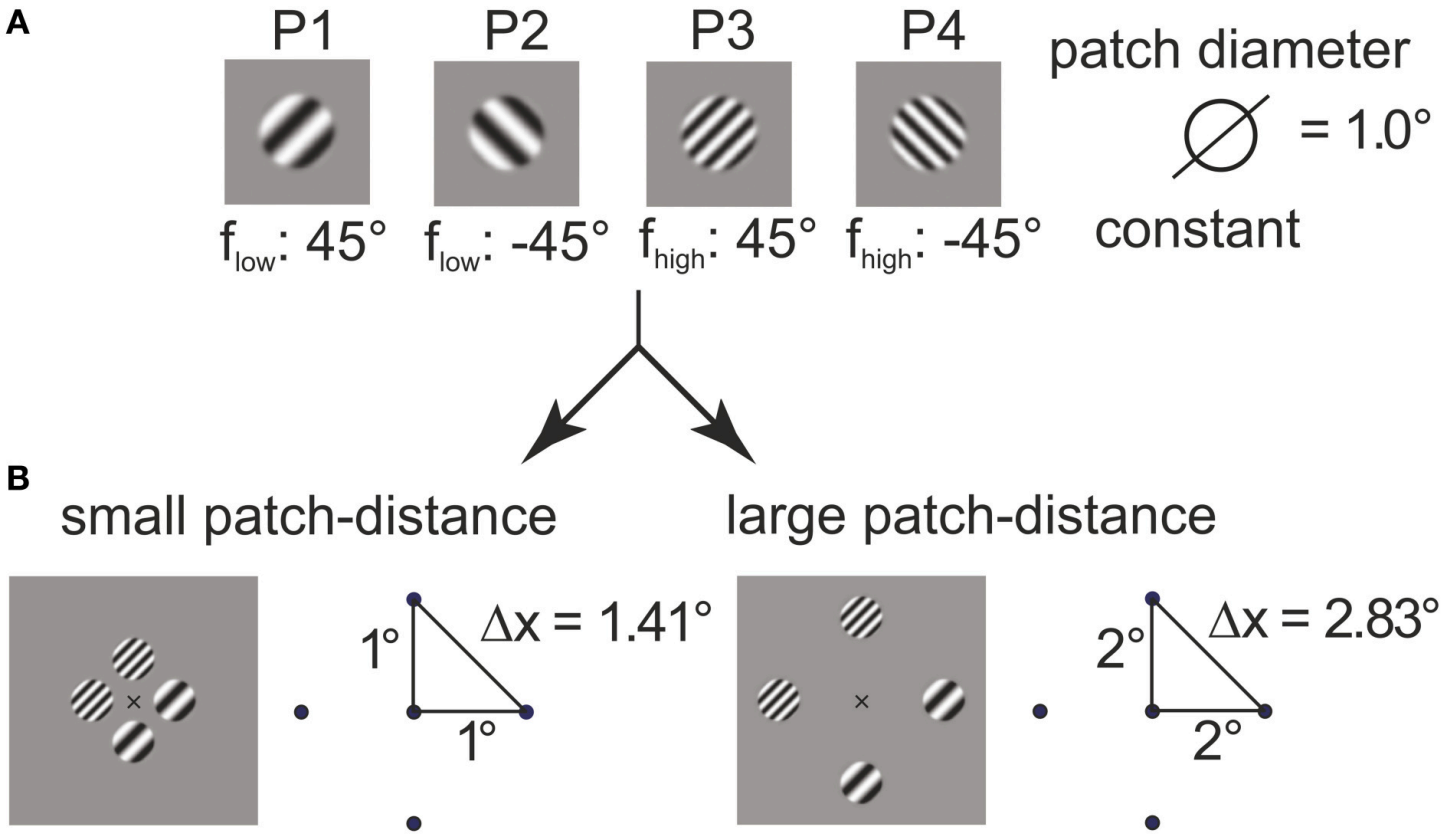
**Construction of plaids from grating patches**. The four grating patches **(A)** used for 4–plaid configurations in small and large inter-patch distance **(B)**.

The grating patches were circular sinusoids with an effective diameter of 1°, achieved by multiplying the sinusoid with a radially symmetric logistic envelope. The envelope was defined as

(1) $$\begin{array}{l} a\left(r\right)=\frac{1+\exp\left(b\left(r-r_{0}\right)\right)}{1+\exp\left(-b\left(r+r_{0}\right)\right)} \end{array}$$

with $r=\sqrt{x^{2}+y^{2}}$ (in degrees of visual angle) and *b* = ln(128)/0.05° (in 1/° of visual angle). The choice for the parameter *b* made the envelope rise (fall, resp.) within the interval (−*r*_0_ − 0.05°, −*r*_0_ + 0.05°) (left) and (*r*_0_ − 0.05°, *r*_0_ + 0.05°) (right), respectively. Two orientations (−45°, 45°) and two carrier spatial frequencies (*f*_low_, *f*_high_) were used, with *f*_low_, *f*_high_ being determined in preparatory measurements (see below). The stimuli are illustrated in Figure 1A (labeled P1–P4).

Grating patches were located on the edge points of the cardinal axes of a spatial position grid to define square arrangements. Two inner radii were were used to define squares with near (1° inner radius, side length ${\sqrt{2}}^{{}^{\circ}}$) and far (2° inner radius, side length ${\sqrt{8}}^{{}^{\circ}}$) patch distance (see Figure 1B).

#### 2.1.3. Stimulus plaid configurations and experimental design

**Figure 2:**
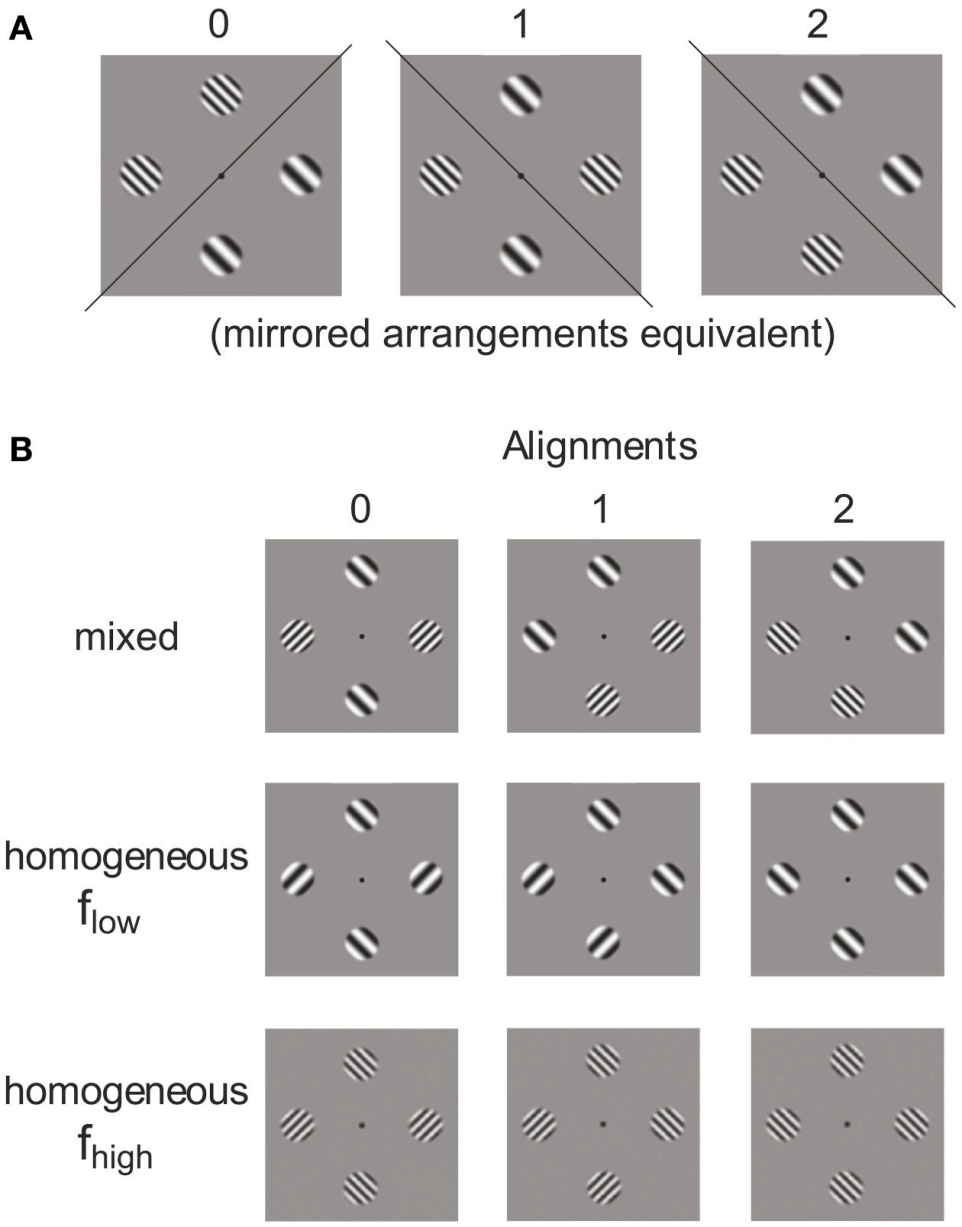
**Categories of plaids**. Different grating patch configurations obtained from allocating two pairs of patches to four locations **(A)** and alignment variation in frequency–homogeneous and inhomogeneous patch compositions **(B)**.

In order to create different patch configurations we first formed pairs of the 4 primary stimuli P1–P4 (i.e., left or right oblique gratings with either high or low spatial frequency). Allowing replication of the same element, $\left(\begin{array}{c} 4\\ 2 \end{array}\right)\;+\;4=10$ pairs can be formed. The pairs were then doubled to create 4-tuples containing one replication of the same element. Such sets can be allocated to 4 locations in 4!/(2!2!) = 6 different ways. However, as illustrated in Figure 2A, the 6 spatial arrangements fall into 3 base configurations, each one having a mirrored equivalent (see mirror axes in Figure 2A). For pairing the same stimuli (i.e., P1-P1, P2-P2, P3-P3, P4-P4) the 3 base configurations are not distinguished. This means there are $3\left(\begin{array}{c} 4\\ 2 \end{array}\right)\;+\;4=22$ distinct spatial arrangements. According to this rule of combining the 10 pairs to the 3 spatial configurations plaid configurations with 0, 1, or 2 orientation alignments of the patches in subsets containing both, only the low and only the high spatial frequency were formed. This means that alignment (0, 1, 2) and spatial frequency homogeneity (mixed, *f*_low_, *f*_high_) are the dimensions of an orthogonal experimental plan for generating plaid configurations from 4 patches with either right or left oblique orientation and either high or low spatial frequency (see Figure 2B).

#### 2.1.4. Subjects

Two male students, FA (22 years) and KF (25 years), served as subjects. Both were highly experienced psychophysical observers and familiar with staircase procedures for contrast detection threshold measurement. Both were paid for participation as part of their student aid contract. Prior to the experiment, participants were informed about the course and expected duration of the experiment. They received a general description of the purpose of the experiment but not about specific outcome expectations. All participants signed a written consent form according to the World Medical Association Helsinki Declaration and were informed that they could withdraw from the experiment at any time without penalty. At the time of data collection, no local ethics committee was instated. Non-invasive experimental studies without deception did not require a formal ethics review provided the experiment complied with the relevant institutional and national regulations and legislation which was carefully ascertained by the authors. After completing the experiment, a summary of their individual data was shown to the observers and the results pattern explained within the scope of the purpose of the study.

#### 2.1.5. Contrast threshold measurement procedure

Contrast thresholds were measured with an adapted version of the method of limits (see Meinhardt, 1999). The method was constructed as a semi-adaptive method that adjusted starting values from the results of former measurements within a set of successive runs, but kept the advantage of multiple independent threshold determinations, as the original limits method. A temporal staircase with a range of 512 equidistant contrast steps, each of which with 35 ms duration, was used. By this procedure we estimated a contrast threshold value in the *i*-th trial, Θ_*i*_, from two up-runs and two down-runs. This was done as follows: the initial contrast was set to the starting value. For the first measurement, this was a value well above threshold, for the subsequent measurements this value was the last threshold contrast measured +25% of contrast. Then the first down-run started: The contrast was decremented using a temporal staircase until the subject signaled that the pattern was no longer visible by pressing a button on a small response keyboard. Then the contrast was diminished by 25% and the contrast was incremented using the temporal staircase until the subject signaled that the pattern was just distinguishable from the background. Then the average of both threshold contrast values Θ_0, *up*_ and Θ_0, *down*_ was taken, and after adding 25% of contrast this value was assigned to the next starting contrast and a second down-up-run started. The contrast threshold Θ_0_ was then computed as the mean of all four threshold determinations. Eight replications of this threshold measurement procedure were carried out for each of the 22 spatial plaid arrangements. All threshold measurements for the plaid patterns were randomly interleaved. The subjects were instructed to rest their judgements on any local deviations of contrast they perceived.

#### 2.1.6. Apparatus

Patterns were programmed using the VSG2/3 stimulus generator (Cambridge Research Systems) and displayed on a EIZO FlexScan 6600 21” grayscale monitor with gamma-correction. The linearity of the digital gray values of the VSG2/3 and luminance *L* in cd/m^2^, measured by an LMT 1003 photometer, was checked before each experimental session. Grating patterns were displayed using a linear gray staircase with 256 entries chosen from a palette of 4096 possible gray values, the medium step (128) always referring to gray value no. 2048. Contrast variation was realized by scaling the step size of the staircase. Hence, independent of contrast a grating was always displayed with a grayscale resolution of 256 steps. We used Maxwell contrast as the contrast metric for the grating plaids, Θ = (*L*_max_ − *L*_min_)/(2*L*_0_). The contrast value describes the contrast of each single patch, while all 4 patches of a plaid had the same contrast. The luminance of the grating patches was modulated across the mean luminance *L*_0_ of the screen, which means that a grating of 0 contrast had mean luminance. The refresh rate of the monitor was 85 Hz at a horizontal frequency of 67.8 kHz, the pixel resolution was set to 1024 × 768 pixels. The room was darkened so that the ambient illumination matched the illumination on the screen. The mean luminance of the screen was set to *L*_0_ = 50 cd/m^2^. Patterns were viewed monocularly at a distance of 75 cm. The subjects used a chin rest and an ocular. The ocular limited the visible area of the screen to a circular field of 8.5° in diameter. A small black dot in the center of the screen was used for fixation. The subjects signaled the presence or absence of the stimulus by pressing a button on an external response box.

#### 2.1.7. Preparation, preliminary measurements, and estimation of threshold reduction

In preliminary measurements the threshold contrasts for a single grating patch, presented on any of the four possible patch positions on the spatial grid, was determined for both the small and large patch distance. Measurements for the two patch distances were arranged in separate experimental blocks. As for the plaid patterns the adapted version of the method of limits was used as the threshold measuring procedure (see above). In order to avoid spatial uncertainty effects (Yager et al., 1984; Hübner, 1996) the fixation point turned into a small arrow that pointed to the grid position where the patch was successively presented. The grid position for stimulus presentation changed randomly from trial to trial. Seven carrier spatial frequencies, ranging from 1.5 to 7 cycles per degree (cpd), were tested. Sixteen replications of the threshold measurement procedure were carried out for each carrier spatial frequency. The threshold contrast as a function of spatial frequency were fitted with a 3rd order polynomial, and two spatial frequencies with equal contrast threshold below and beyond the minimum were extrapolated. These frequencies were *f*_low_ = 2cpd, *f*_high_ = 4cpd for subject FA and *f*_low_ = 2cpd, *f*_high_ = 5cpd for subject KF. For both subjects, the contrast threshold functions for the small and the large patch distance were shifted against each other on the contrast scale but the principal course across spatial frequency was the same. The subject specific selections for *f*_low_ and *f*_high_ were used for constructing the plaid stimuli in the main experiment.

The average threshold contrast (across trials and subjects) for the two equally detectable spatial frequency patches were Θ_0_ = 0.0111 for the small distance grid (1°) and Θ_0_ = 0.0166 for the large distance grid (2°).

In order to judge whether a given plaid configuration caused inhibitory or excitatory patch interactions across the four grid positions the expected threshold contrast for the assumption of spatial independence was derived from the threshold contrast for a single grating patch. Assuming probability summation (detailed derivations see Supplementary Material) yields estimated threshold reduction factors ${\hat{\Theta }}_{0}$ between 0.673 and 0.707. Multiplying Θ_0_ with ${\hat{\Theta }}_{0}$ yields the threshold contrast prediction for probability summation on the non-normalized scale.

#### 2.1.8. Data analysis

The threshold contrast mean across all trials for the same condition was used as the estimate of the true threshold contrast for each plaid configuration. The threshold contrast means of the two subjects were again averaged to result in the contrast threshold estimate for the *c*-th plaid configuration, ${\bar{\Theta }}_{c}$. Since there were *n* = 8 replications of contrast threshold measurement for each plaid configuration, ${\bar{\Theta }}_{c}$ rested on *M* = 2*n* replications. Standard errors $s_{e}\left({\bar{\Theta }}_{c}\right)=\sigma \left(\Theta_{c}\right)/\sqrt{M}$, were based on pooled variance estimates from the data of the two subjects, $\sigma^{2}\left(\Theta_{c}\right)=\left(ns_{c,1}^{2}+ns_{c,2}^{2}\right)/\left(2n-2\right)$. Confidence intervals for ${\bar{\Theta }}_{c}$ were calculated assuming a Student *t*-distribution for the means, $CI={\bar{\Theta }}_{c}\pm t_{\left(0.975;M-1\right)}s_{e}\left({\bar{\Theta }}_{c}\right)$. The critical test for the *c*-th plaid configuration was to decide whether the contrast interval predicted by probability summation among 4 grating patches fell beyond, below, or within the confidence interval of the threshold contrast mean, ${\bar{\Theta }}_{c}$.

### 2.2. Cortex model

#### 2.2.1. Outline

We studied two variants of a recurrently coupled, neuronal network model representing populations in early visual cortex engaged in processing plaid stimuli. Recurrent weights were adapted such that network activations for different plaid configurations most closely predicted human detection thresholds. The structure of the model was held as simple as possible, for having a minimum number of free parameters while still being able to reproduce all experimental findings.

#### 2.2.2. Single units

Each unit *i* in our model network represents a population of neurons and is described in terms of its mean activity *A*_*i*_(*t*). Activation changes in dependence on the current feedforward input $J_{i}^{\text{ffw}}$(*t*) and recurrent feedback $J_{i}^{\text{rec}}$(*t*), and is described by a time coarse-grained Wilson-Cowan dynamics (Wilson and Cowan, 1972)

(2) $$\tau \frac{dA_{i}(t)}{dt}=-A_{i}(t)+g\;[J_{i}^{\text{rec}}(t)+J_{i}^{\text{ffw}}(t)-J^{\text{thr}}]\;.$$

**Figure 3:**
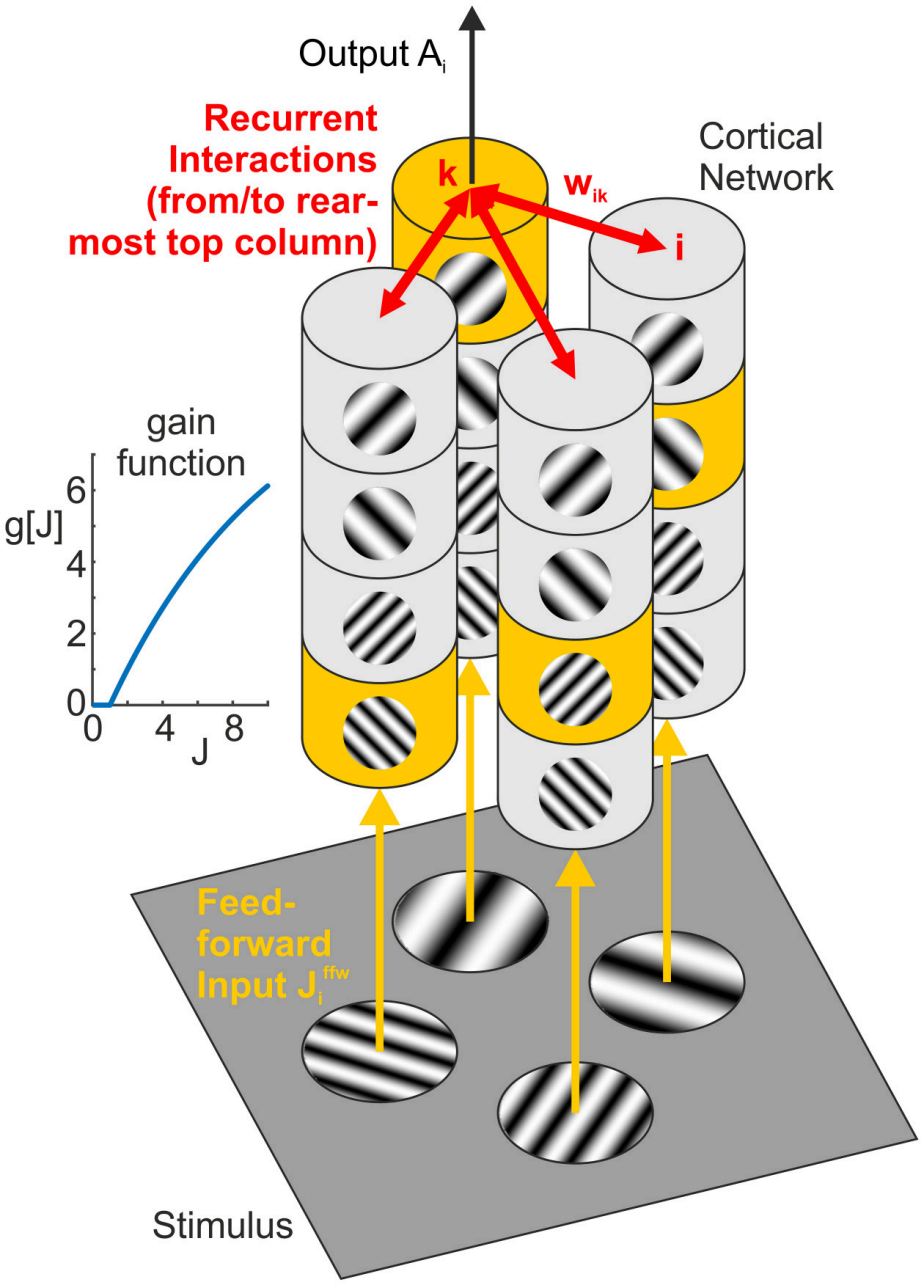
**Network model**. Feedforward input from the visual stimulus (bottom) activates neural columns (marked in yellow) with matching orientation and spatial frequency (SF) preference in each of the four hypercolumns (vertical structures). Horizontal interactions (in red) provide recurrent feedback between different (hyper-)columns in the network. Note that for clarity, we only show connections originating from the top column in the rearmost hypercolumn, targeting columns with the same orientation and SF preference in the neighboring three hypercolumns (i.e., the set of interactions shown in the top left subpanel of **Figures 6**, **7**). The inset graph shows the neural gain function *g*[*J*] mapping a synaptic input *J* to a neural population response.

Here, τ is a time constant (w.l.o.g. set to 1), and *g*[…] denotes a rectifying gain function which we choose to be *g*[*J*]: = *J*^max^(1 − ((*J*^max^ − 1)/*J*^max^)^*J*^) for *J* > 0 and 0 otherwise. Choosing *J*^max^ = 10, the gain function is approximately linear with slope 1 for *J* = 1 and saturates at *J*^max^ for *J* → ∞ (inset Figure 3). For simplicity, we model *A* as a dimensionless quantity which can, for an intended comparison to a particular experimental situation, be scaled to fit the corresponding neurophysiological quantity such as the population firing rate.

#### 2.2.3. Full network

The network consists of *i* = 1, …, *N* = 16 units, comprising four “hypercolumns” of four units each (Figure 3, vertical structures). The four units in each hypercolumn represent populations with different preferred orientations and preferred spatial frequencies, but with same spatial (classical) receptive field centered on one of the four positions within a plaid configuration. For a specific plaid configuration, exactly one unit in each hypercolumn becomes activated with an input of *J*^ffw^ > 0. All other units receive a feedforward input of *J*^ffw^ = 0 leading to zero activation, which is a simplification of the fact that neurons with receptive field properties deviating from or being orthogonal to the properties of the stimulus are only weakly activated or remain silent, respectively.

Recurrent input $J_{i}^{\text{rec}}$ provides feedback from other units via a coupling matrix *W* = {*w*_*ik*_}, $J_{i}^{\text{rec}}\left(t\right)=\underset{k}{\sum }w_{ik}A_{k}\left(t\right)$ (w.l.o.g. we assume self-interactions to be zero). For finding suitable weights, we used two complementary approaches. These have different advantages and disadvantages as explained below.

#### 2.2.4. No prior assumptions on interactions

In our first approach (from here on termed “model A”), we decided to ignore prior knowledge about the nature of interactions from psychophysical or physiological evidence. Having this (essentially) assumption-free approach allows discovering functional principles going beyond the current state of anatomical and functional knowledge. The high number of degrees-of-freedom (*df*'s) can be drastically reduced by imposing symmetry constraints to the weights (details see Supplementary Material), leading to 30 free parameters.

#### 2.2.5. Postulating interactions from prior knowledge

In our second approach (“model B”), we computed weights from postulating three types of (parametrized) interactions which were motivated from psychophysical or physiological evidence (Polat and Sagi, 1993, 1994; Kapadia et al., 2000). Although being more restrictive on “weight space,” this approach yields a parametrization of interactions that can be extended to other stimuli, such as more complex plaid configurations going beyond (2 × 2)-patches.

In particular, we hypothesized that three types of interactions play a role for explaining contextual integration:

- *w*^iso^: Orientation-*unspecific*, isotropic *inhibitory* interactions
- *w*^ori^: Orientation-*specific, excitatory* interactions (between *similar* spatial frequencies)
- *w*^frq^: Orientation-*specific, inhibitory* interactions (between *different* spatial frequencies).

All of these types have a typical strength and range of interaction, described by Gaussian functions with free parameters amplitude, mean, and variance, giving a total of 9 *df* as compared to the 30 *df* in model A. The total interaction strength *w*_*ik*_ between units *i* and *k* is then obtained by adding these three contributions, $w_{ik}=-w_{ik}^{\text{iso}}+w_{ik}^{\text{ori}}-w_{ik}^{\text{frq}}$.

#### 2.2.6. Linking hypothesis

For linking simulation to experiment, we needed a suitable mapping of model activities to psychophysical detection thresholds Θ. In general, we assumed that the *higher* model activity gets with a *fixed* input, the lower will be the corresponding detection threshold. This assumption is equivalent to the required input becoming *lower* in order to achieve a *fixed* activation level. The reciprocal dependency between activity *A* and average human threshold $\bar{\Theta }$ does not need to be linear, but can be convex or concave. With setting *A*^min^: = 0, introducing two additional free parameters *A*^max^ and κ, and abbreviating total activity in the steady state as $A^{\infty }=\underset{i}{\sum }A_{i}\left(t\to \infty \right)$, we defined a linking hypothesis by

(3) $${\hat{\Theta }}_{c}:=\;\Theta^{\text{min}}+(\Theta^{\text{max}}-\Theta^{\min}){\left(\frac{A_{c}^{\infty }-A^{\min}}{A^{\max}-A^{\min}}\right)}^{\kappa }.$$

Here, Θ^min^ and Θ^max^ are the minimum and maximum thresholds measured in the experiment, respectively, while *c* indexes the plaid configuration for which the corresponding threshold ${\bar{\Theta }}_{c}$ was measured. By Equation (3), ${\hat{\Theta }}_{c}$ defines the model estimate for ${\bar{\Theta }}_{c}$.

### 2.3. Natural image statistics

**Figure 4:**
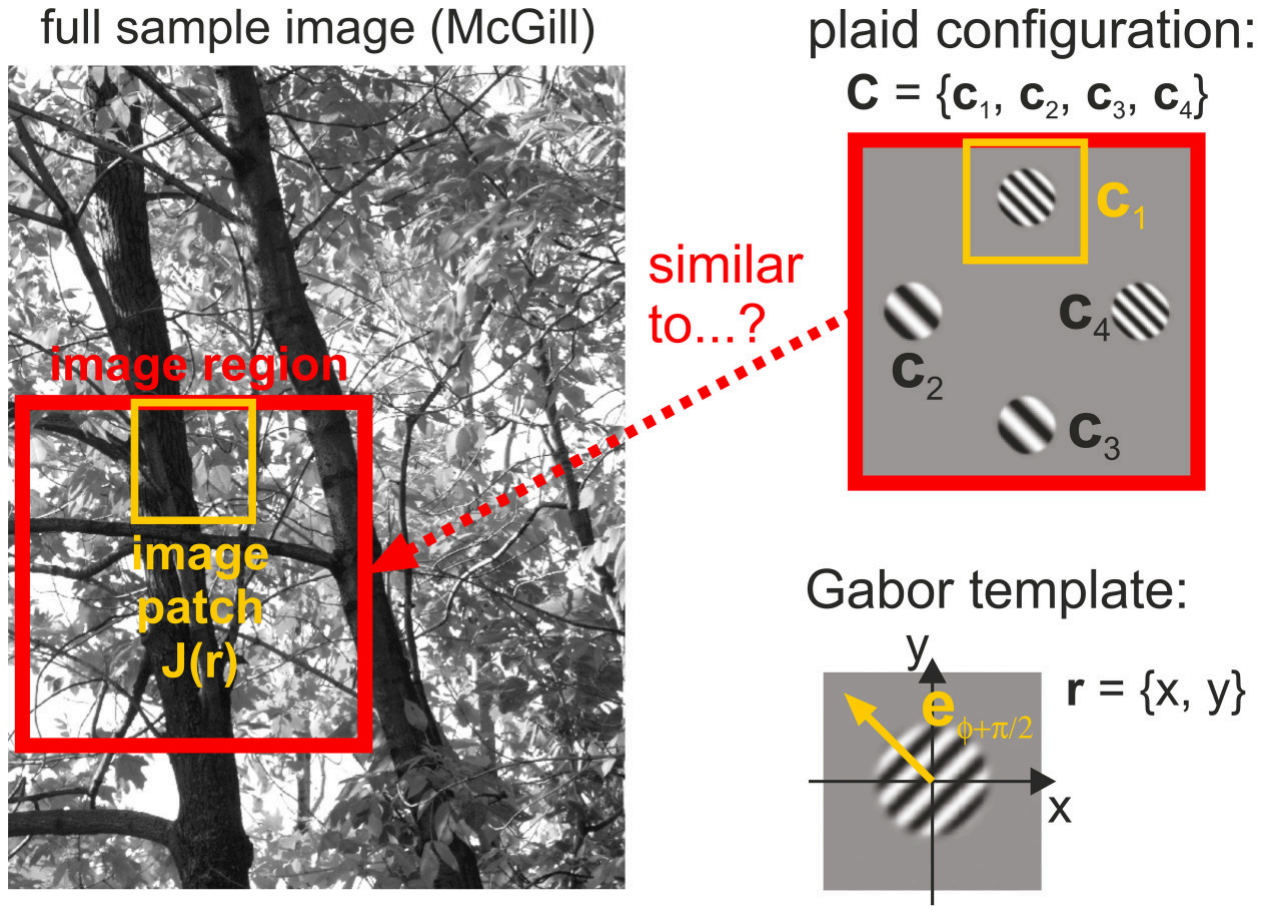
**Image analysis**. Image regions taken from a full image converted to gray scale (left) are compared with plaid configurations (top right) by comparing Gabor templates (bottom right) with different spatial frequencies and different orientations to image patches (yellow outlines) positioned at the four positions in a plaid.

We also performed a natural image analysis to test our hypothesis that human detection thresholds are linked to the frequency with which different plaid configurations occur in natural scenes. For this purpose, we quantified whether plaids occur more or less often than predicted from the likelihoods of their constituting single patches. This statistics was derived from analyzing how similar local image regions are to the four different oriented gratings used in our experiments (Figure 4). Mathematical details of the procedures described below can be found in the Supplementary Material.

Image processing consisted of the following basic steps, hereby making use of the convolution theorem to realize whitening and Gabor filtering in a numerically efficient way:

1. Conversion from RGB color space to grayscale.
2. Transformation into Fourier space.
3. Multiplication by Whitening filter *F*_*w*_(**k**), see below.
4. Multiplication by Gabor filter(s) *g*_**p**_ transformed into Fourier space.
5. Inverse Fourier transform, thus providing the grating patch – image patch overlaps *O*_**p**_(**r**) for each position **r** in the image, see below.
6. Removal of an image border of width 4σ (which is approximately the size of one Gabor template) for excluding Fourier transformation artefacts at the (non-periodic) image boundaries.

In natural scenes, lower spatial frequencies typically occur with higher amplitudes than higher spatial frequencies (van der Schaaf and van Hateren, 1996). To compensate for this effect, whitening is used to equalize the average spectral composition of image ensembles, allowing us to separate the actual probabilities of occurrence of the different grating plaid configurations from the typical intensity with which they are present.

The grating patch – image patch overlaps *O*_**p**_ statistically quantify how well an image patch is explained by the presence of a single grating with parameters **p** (e.g., comprising orientation and spatial frequency, detailed explanation see Supplementary Material). Consequently, it also allows assessing the presence or absence of full plaid configurations **C** comprising a combination of four grating patches. To quantify how often configuration **C** is encountered in an image ensemble E, we computed the ratio Λ(**C**) between the joint likelihood to observe **C** and the likelihood to independently observe the single grating patches **c**_*i*_ of **C**:

(4) $$\begin{array}{rcl} \Lambda \left(\text{C}\right) & := & \frac{L\left(\text{C}|E\right)}{\overset{4}{\underset{i=1}{\prod }}L\left({\text{c}}_{i}|E\right)} \end{array}$$

For example, a value of Λ(**C**) = 2 would mean that plaid **C** occurs twice as frequently as expected from the probability of occurrence of its single grating patches **c**_*i*_.

As image ensembles E, we used two different data bases: first, the Corel Image Database [Corel Mega Gallery (add-on to CorelDraw version 6), Corel Corporation (1996)] with about 68,000 images of size 384 × 256 pixels in JPEG-compression, and the McGill Color Image Database with about 820 color-calibrated images of size 576 × 768 pixels without compression (Olmos and Kingdom, 2004). JPEG compression is known to introduce artifacts in cardinal orientations. However, since we were only interested in oblique orientations this putative confounding factor was of negligible concern for our investigations.

## 3. Results

### 3.1. Experiment

**Figure 5:**
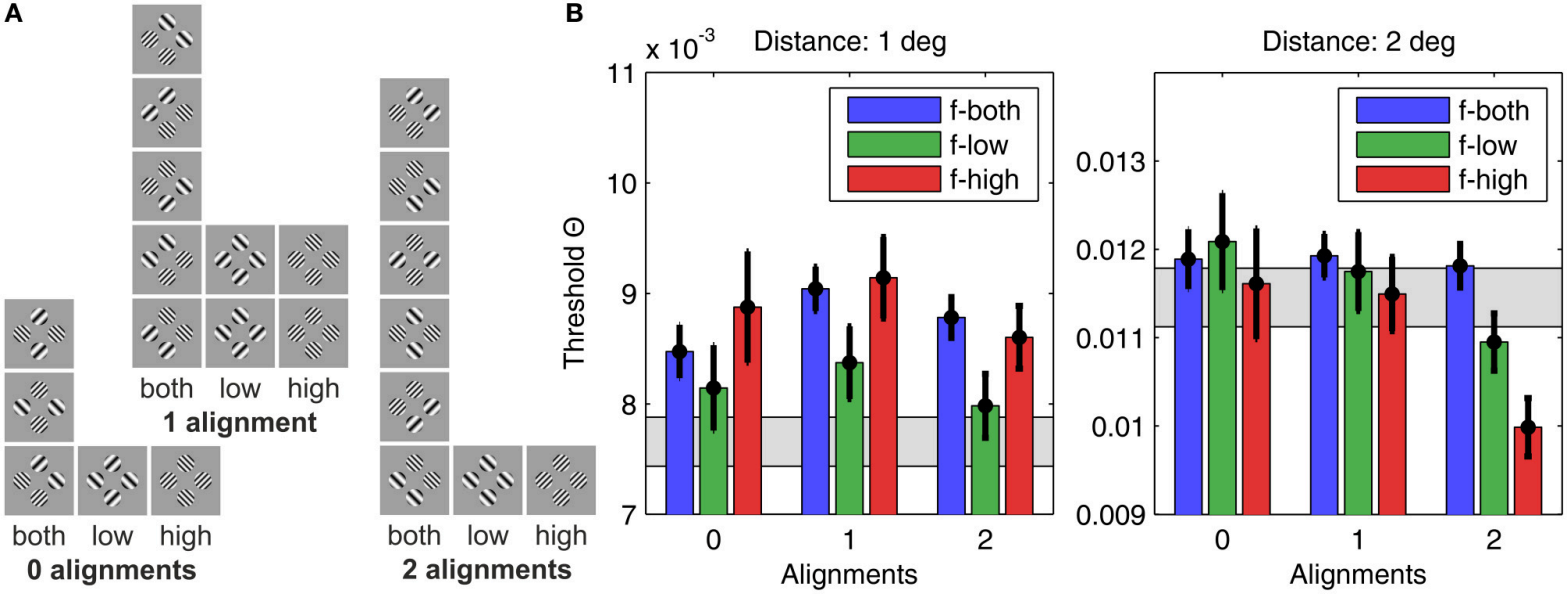
**Human detection thresholds for different plaid configurations**. **(A)** The 22 plaid configurations used in the experiment, sorted according to grating patch alignment (zero alignments, one alignment, or two alignments) and SF content (only low SFs, only high SFs, or both SFs), giving nine categories in total. **(B)** The graph on the left shows results for plaid distance *d* = 1°, and the graph on the right for plaid distances of *d* = 2°. The height of the bars indicates the average detection threshold and the vertical black lines the corresponding 95% confidence interval. For comparison, the gray bars display the approximate detection thresholds predicted from single element detection by assuming independency and probability summation. Bars significantly above the gray region thus indicate suppressive interactions, while bars significantly below indicate facilitating interactions.

The threshold contrast results are summarized in Figure 5B for the small distance grid (1°, left panel) and the large distance grid (2°, right panel). The results patterns for small and larger inter-patch distance were remarkably different. To substantiate different effects of alignment and spatial frequency for the two grid sizes we analyzed the threshold contrast data with ANOVA, and tested against the assumption of probability summation among the four grid positions with a confidence interval test.

#### 3.1.1. Results for the small distance grid (1°)

For the small distance grid there were main effects of alignment [*F*_(2, 875)_ = 8.32, *p* < 0.001] and spatial frequency [*F*_(2, 875)_ = 13.64, *p* < 0.001], but no significant interaction of both factors [*F*_(4, 875)_ = 1.79, *p* = 0.129]. Pairwise comparisons showed that configurations with 1 alignment had significantly larger threshold contrasts compared to configurations with 2 alignments [*F*_(1, 875)_ = 16.31, *p* < 0.001] and 0 alignment [*F*_(1, 875)_ = 5.62, *p* < 0.02], while threshold contrasts did not differ significantly for 0 and 2 alignments [*F*_(1, 875)_ = 0.517, *p* = 0.517]. Homogeneous low spatial frequency patches were detected at lower contrasts than homogeneous high frequency patches [*F*_(1, 875)_ = 20.61, *p* < 0.001], and also compared to plaids combining both spatial frequencies [*F*_(1, 875)_ = 22.95, *p* < 0.001]. High spatial frequency and mixed frequency plaids were detected at equal contrast levels [*F*_(1, 875)_ = 0.44, *p* = 0.506].

The hypothesis of probability summation among equally detectable grating patch stimuli at the 4 grid positions was tested with a confidence interval test for the threshold contrast derived from assuming probability summation, using estimates of the shape parameter β in the interval [3.5, 4] (see Supplementary Material). For this range of β, ${\hat{\Theta }}_{0}=k^{-1/\beta }$ leads to estimated threshold reduction factors in the range of [0.673, 0.707]. For the measured threshold contrast of a single patch at any of the 4 grid positions, Θ_0_ = 0.0111, the threshold contrast for probability summation among 4 grating patches presented simultaneously on the spatial grid is expected within the interval [0.0075, 0.0078] (see gray shaded area in Figure 5B, left panel; see Table 1). The test shows that only plaid configurations with low spatial frequencies at 0 and 2 alignments had threshold contrasts that were compatible with a probability summation mechanism. All the other combinations yielded threshold contrasts significantly above the prediction, indicating strong inhibitory interactions. For 1 alignment, where the orientations of 2 grating patches were orthogonal to an aligned array of the 2 other gratings, inhibitory interactions were strongest, and present for all spatial frequency compositions.

**Table 1:** **Confidence interval tests for 1°**.

**Alignments**	**Spatial frequency**	**$\bar{\Theta }$**	***s*_*e*_**	**Θ_*l*_**	**Θ_*u*_**	**CI(β_1_)**	**CI(β_2_)**
0	*f*_low_	0.0081	0.0002	0.0077	0.0086	OUT	IN
1	*f*_low_	0.0084	0.0002	0.0080	0.0087	OUT	OUT
2	*f*_low_	0.0079	0.0001	0.0077	0.0082	OUT	IN
0	*f*_high_	0.0089	0.0002	0.0084	0.0094	OUT	OUT
1	*f*_high_	0.0091	0.0002	0.0088	0.0095	OUT	OUT
2	*f*_high_	0.0085	0.0001	0.0082	0.0087	OUT	OUT
0	*f*_both_	0.0084	0.0001	0.0082	0.0087	OUT	OUT
1	*f*_both_	0.0090	0.0001	0.0088	0.0092	OUT	OUT
2	*f*_both_	0.0088	0.0001	0.0086	0.0090	OUT	OUT

Confidence interval tests for the prediction of detection by probability summation among the 4 grid positions for the small distance grid (1°). The table shows mean, $\bar{\Theta }$, standard error, s_e_, lower and upper confidence limits for a 5% α-level, Θ_l_ and Θ_u_, and decision whether the probability summation prediction falls within or outside the confidence interval of the mean, assuming β_1_ = 3.5 and β_2_ = 4.0.

#### 3.1.2. Results for the large distance grid (2°)

For the large distance grid there were main effects of alignment [*F*_(2, 875)_ = 19.65, *p* < 0.001] and spatial frequency [*F*_(2, 875)_ = 12.04, *p* < 0.001], and also the interaction of both factors reached significance [*F*_(4, 875)_ = 5.78, *p* < 0.001]. The data shown in the right panel of Figure 5B confirm that the main effects of spatial frequency and alignment were not unique, but mediated by the alignment × spatial frequency interaction. Pairwise comparisons across alignment revealed that, for mixed spatial frequencies, there was no alignment effect [D(0–1): *F*_(1, 875)_ = 0.03, *p* = 0.873; D(0–2): *F*_(1, 875)_ = 0.11, *p* = 0.745; D(1–2): *F*_(1, 875)_ = 0.33, *p* = 0.567]. For spatial frequency homogeneous plaid configurations the threshold contrasts did not differ for 0 and 1 alignment, but were significantly lowered for 2 alignments, with a particularly pronounced threshold reduction at 2 alignments for homogeneous high spatial frequency plaids [*f*_low_: D(0–1): *F*_(1, 875)_ = 0.71, *p* = 0.398; D(0–2): *F*_(1, 875)_ = 9.21, *p* < 0.01; D(1–2): *F*_(1, 875)_ = 7.35, *p* < 0.01; *f*_high_: D(0–1): *F*_(1, 875)_ = 0.08, *p* = 0.772; D(0–2): *F*_(1, 875)_ = 18.81, *p* < 0.001; D(1–2): *F*_(1, 875)_ = 25.26, *p* < 0.001].

The confidence interval test for probability summation among grating patches at the four grid positions showed that the probability summation hypothesis could not be rejected for all plaid configurations with 0 and 1 alignment (see gray shaded area in Figure 5B, right panel; see Table 2). For 2 alignments, probability summation was compatible with the threshold contrast data for mixed frequency plaids. For high spatial frequency plaids, the threshold contrasts fell significantly below the predicted contrast range. For low spatial frequency plaids, the confidence interval of threshold contrasts fell significantly below the predicted contrast range for larger β values, but had an intersection with predicted threshold contrasts when smaller β values were assumed.

**Table 2:** **Confidence interval tests for 2°**.

**Alignments**	**Spatial frequency**	**$\bar{\Theta }$**	***s*_*e*_**	**Θ_*l*_**	**Θ_*u*_**	**CI(β_1_)**	**CI(β_2_)**
0	*f*_low_	0.0121	0.0003	0.0114	0.0127	OUT	IN
1	*f*_low_	0.0117	0.0002	0.0113	0.0122	OUT	IN
2	*f*_low_	0.0110	0.0002	0.0106	0.0113	IN	OUT
0	*f*_high_	0.0116	0.0003	0.0110	0.0123	IN	IN
1	*f*_high_	0.0115	0.0002	0.0110	0.0119	IN	IN
2	*f*_high_	0.0100	0.0002	0.0096	0.0103	OUT	OUT
0	*f*_both_	0.0119	0.0002	0.0115	0.0123	OUT	IN
1	*f*_both_	0.0119	0.0001	0.0116	0.0122	OUT	IN
2	*f*_both_	0.0118	0.0001	0.0116	0.0121	OUT	IN

Confidence interval tests for the prediction of detection by probability summation among the 4 grid positions for the large distance grid (2°). Conventions as in Table 1.

The overall picture of threshold contrast results differed remarkably for the two distance grids. For the small grid there was evidence for inhibitory interactions, being most pronounced for the 1 alignment configurations. For the large grid there was evidence for independence, except for frequency homogeneous configurations. These reflected excitatory interactions, being much better visible than predicted by an OR-detection rule for spatially distributed stimulus events.

### 3.2. Cortex model

For assessing how well a model with a particular set of adapted parameters fits the experimental data, we first counted the number of conditions *N*_outside_ in which the model prediction fell outside the confidence intervals around the measured thresholds. The lower the minimum *N*_outside_ achieved over the full set of simulations, the better the fit between model and experiment. In total, there were 19 conditions: 9 plaid configurations for each distance plus one probability summation threshold which we required the model to reproduce if all interactions were set to zero (see Supplementary Material). Second, we computed the mean quadratic error *E*_2_ between predicted and measured thresholds.

**Figure 6:**
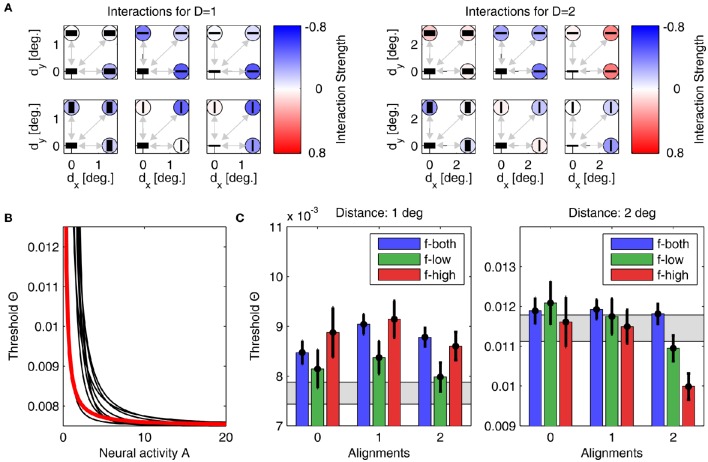
**Parameters and performance of model A**. For this figure, we used the parameter set yielding model results best matching the experimental data. **(A)** Interactions for plaid distances *d* = 1° (left) and *d* = 2° (right). The six subplots corresponding to each plaid distance show interactions between the neuronal unit in the lower left corner to all other units, with their orientation preferences and SFs indicated by the black bars (thick bar for low SF, thin bar for high SF). Interaction strength is color coded (insides of circles). The upper row displays all interactions for units with similar orientation preferences, while the lower row displays interactions between units with orthogonal orientation preferences. The outer columns display interactions between units with similar SFs, while the middle column displays interactions between units with different SFs. For simplifying the figure, the original plaid configuration has been rotated by 45 degrees. **(B)** Mapping of activities onto thresholds for the 'best' model (thick red line), and for nine other models with the next-best performances (thin black lines). **(C)** Comparison of detection thresholds from model and experiment for plaid distances *d* = 1° (left) and *d* = 2° (right). The predicted thresholds from the model are displayed as colored bars (color code as inset), while the psychophysical thresholds are indicated by the black circles with the vertical lines showing the corresponding 95%-confidence intervals. The region shaded in gray color indicates the range of thresholds expected from probability summation. Note that model predictions and actual thresholds are indistinguishable from each other.

Parameter optimization of model *A* was performed starting from 500 different initial conditions. After convergence of the stochastic gradient descent method, 457 parameter sets yielded thresholds for which *N*_outside_ = 0, giving an average *E*_2_ of 1.68 · 10^−6^ ± 4.72 · 10^−6^. The best performing model with lowest quadratic distance *E*_2_ = 6.6 · 10^−8^ is shown in Figure 6, with its interactions displayed in panel Figure 6A, and the corresponding linking function in Figure 6B. Detection thresholds are shown in panel Figure 6C, demonstrating a perfect fit between model and experiment. In particular, this fit is much closer than the human response variability in the experiment expressed by the confidence intervals. Typically, such a perfect match indicates that a model contains too many free parameters (“overfitting”) and thus will not generalize well to other experimental situations. For different initializations, the parameters after convergence were very similar, which we demonstrate by also showing the nine linking functions for the next best matches of model to experiment (Figure 6B, black lines). All linking functions have exponents around 1.17–3.35 and exhibit a similar shape (concave down).

**Figure 7:**
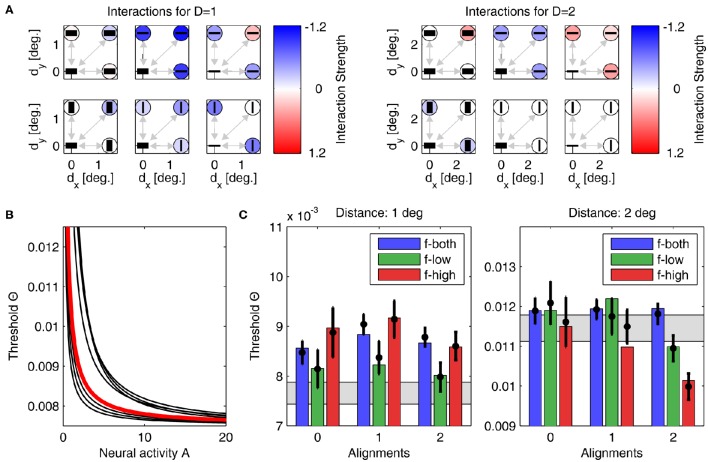
**Parameters and performance of model B**. We again use the parameter set yielding the best matching results. Display as in Figure 6. Although match of model to experiment is not as good as before, still only one model prediction is outside the confidence interval.

For model B, we also performed simulations from 500 initial conditions. Since the lower number of parameters restricts the degrees-of-freedom in the model's dynamics, the 8 best models yielded a minimum *N*_outside_ = 2, with an average *E*_2_ of 2.54 · 10^−4^ ± 4.64 · 10^−5^. The best performing model with lowest quadratic distance *E*_2_ is shown in Figure 7, with its interactions displayed in panels Figure 7A, and the corresponding linking function in Figure 7B. Detection thresholds shown in panels Figure 7C confirm that the fit of model to experiment is now less accurate. However, the number of parameters is about three times lower and thus a perfect fit is less likely than for model A. Again, for different initializations, the parameters after convergence are very similar. For example, the exponent of the linking function now varies between 0.99 and 1.37 (see Figure 7B, black lines, for examples of linking functions). An advantage of model B is that the parametric definition of the interactions as distance-dependent Gaussian functions allows one to predict interaction strengths also for other plaid configurations not used in this particular experiment.

Although individual weight values were different between models A and B, the general pattern which emerged after learning was very similar and thus confirmed the consistency of our approach. Comparing interaction weights for models A and B we find very similar structures that provide an intuitive explanation for the empirical results:

- First, we computed the mean over all interactions as displayed in Figures 6A,B for *d* = 1° and *d* = 2°. Averaged over the 457 (8) best models of type A (type B), we obtained 〈*w*〉 = −0.14 ± 0.07 for 1° and 〈*w*〉 = −0.03 ± 0.02 for 2° (〈*w*〉 = −0.28 ± 0.10 for 1° and 〈*w*〉 = −0.01 ± 0.05 for 2°), respectively. Clearly, for the smaller patch distance interactions must be more inhibitory, thus explaining the higher detection thresholds.
- Second, we assessed the difference in coupling strengths between feature detectors for similar spatial frequencies (low-low or high-high) and coupling strengths between feature detectors for different spatial frequencies (low-high), averaged over patch distances (Table 3). We found that coupling strengths are similar between feature detectors with orthogonal orientation preferences (second and fourth line in Table 3). However, for parallel orientation preferences, interactions between units with different spatial frequency preferences are much lower (inhibitory) than between units with similar preferred spatial frequencies (first and third line in Table 3). These inhibitory couplings explain the higher detection thresholds for plaids with different spatial frequencies.
- Third, we compared the coupling strengths between units with low spatial frequency preferences and units with high spatial frequency preferences, averaged over (relative) orientations (Table 4). Averaged over the best models of type A (type B), we found an inverse relation for different patch distances. In particular for smaller distances, low-frequency interactions must be stronger than high-frequency interactions, while high-frequency interactions must be more positive than low-frequency interactions for larger patch distances.

**Table 3:** **Comparison of interactions between parallel and orthogonal orientation preferences**.

**Model**	**Orientations**	**Low-low SFs**	**High-low SFs**	**High-high SFs**
A	Parallel	+0.01 ± 0.01	−0.24 ± 0.11	+0.06 ± 0.02
	Orthogonal	−0.12 ± 0.05	−0.08 ± 0.04	−0.15 ± 0.05
B	Parallel	+0.05 ± 0.003	−0.55 ± 0.36	+0.08 ± 0.01
	Orthogonal	−0.13 ± 0.02	−0.13 ± 0.02	−0.19 ± 0.03

**Table 4:** **Comparison of interactions between similar spatial frequencies (SFs) for small and large patch distances**.

**Model**	**Distance**	**Low-low SFs**	**Relation**	**High-high SFs**
A	*d* = 1°	−0.10 ± 0.04	>	−0.14 ± 0.07
	*d* = 2°	−0.01 ± 0.03	<	+0.06 ± 0.01
B	*d* = 1°	−0.07 ± 0.02	>	−0.28 ± 0.05
	*d* = 2°	−0.004 ± 0.01	<	+0.17 ± 0.01

### 3.3. Natural image statistics

**Figure 8:**
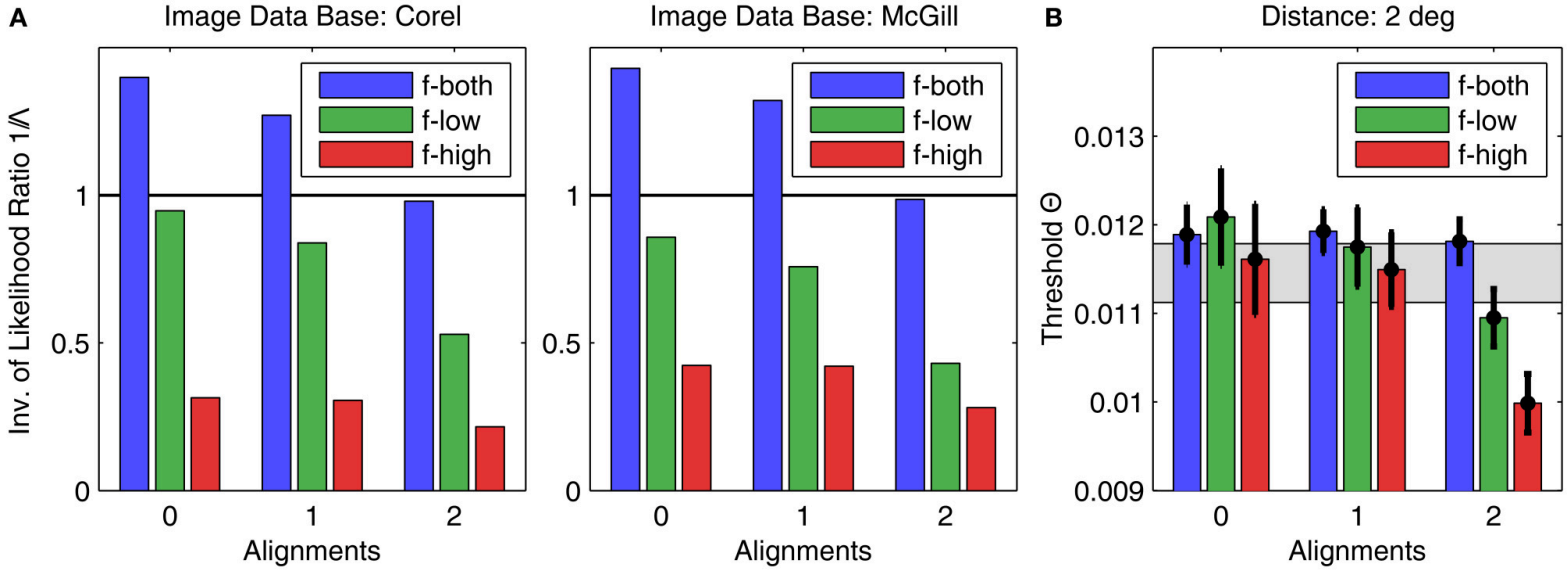
**Results of image analysis compared to human psychophysics**. **(A)** The height of the bars indicates the inverse of the average likelihood ratio Λ for the corresponding plaid configurations, sorted into the same categories as used in Figure 5 for showing the psychophysics results. The left graph and right graphs show the results for the Corel and McGill data bases, respectively. For comparison, **(B)** shows the experimental results for *d* = 2° (same as in Figure 5, right graph), which come closest to the observed result pattern.

The image analysis was performed for all 256 possible patch configurations, including plaids never used in the experiment. For comparison with the psychophysical data, the corresponding likelihood ratios Λ(**C**) for the 22 patch configurations **C** used were extracted and sorted into the 9 categories defined by alignment (0, 1, or 2 alignments) and spatial frequency (only low SFs, only high SFs, or both SFs), identically to the presentation of the experimental data in Figure 5. In Figure 8A, the results are shown for both data bases for a patch distance of *d* = 2°. In particular, we plotted 1/Λ since our hypothesis is that the more likely a patch, the lower will be the corresponding detection threshold. Compared to the experimental data for the same distance shown in Figure 8B, it turns out that the general result pattern is well reproduced, in particular for configurations with two alignments. Two exceptions are the inverse likelihood ratios for high SF configurations: they are much lower than in the corresponding psychophysical data.

Since natural images can be observed from different viewing angles, one degree of visual angle can correspond to a varying numbers of pixels. To check how strongly results depend on this unknown variable, we analyzed image data using a range from 6 to 24 pixels/degree for the Corel data base, and 12–48 pixels/degree for the McGill data base, over a range of spatial distances from *d* = 0.5° to *d* = 4°. Although results varied quantitatively, the general pattern as displayed in Figure 8 remained unchanged (not shown). There was also no conspicuous change when we varied the threshold used for reducing noise. While this finding means that the psychophysical data for *d* = 1° has no apparent relation to natural image statistics, it nevertheless confirms the well-known fact that natural image statistics is in many aspects scale-invariant (Ruderman, 1997).

## 4. Discussion

Combining image analysis, computational modeling and psychophysical experiment, we have investigated visual feature integration of oriented patch gratings and established a link between stimuli (image statistics), predicted brain activity (network model), and cognitive states (perception). In particular, our model consistently and precisely reproduces human detection thresholds in all experimental conditions. Moreover, image statistics are closely linked to perception for 2° inter-patch distance: the more likely a particular plaid configuration, the lower its detection threshold. The model predicts three types of interactions required to explain the observed effects: medium-range spatially isotropic inhibition, long-range iso-orientation excitation for feature detectors with *similar* spatial frequency preferences, turning into suppression between iso-oriented feature detectors with *different* spatial frequencies.

### 4.1. Interactions and their putative computational role

**Figure 9:**
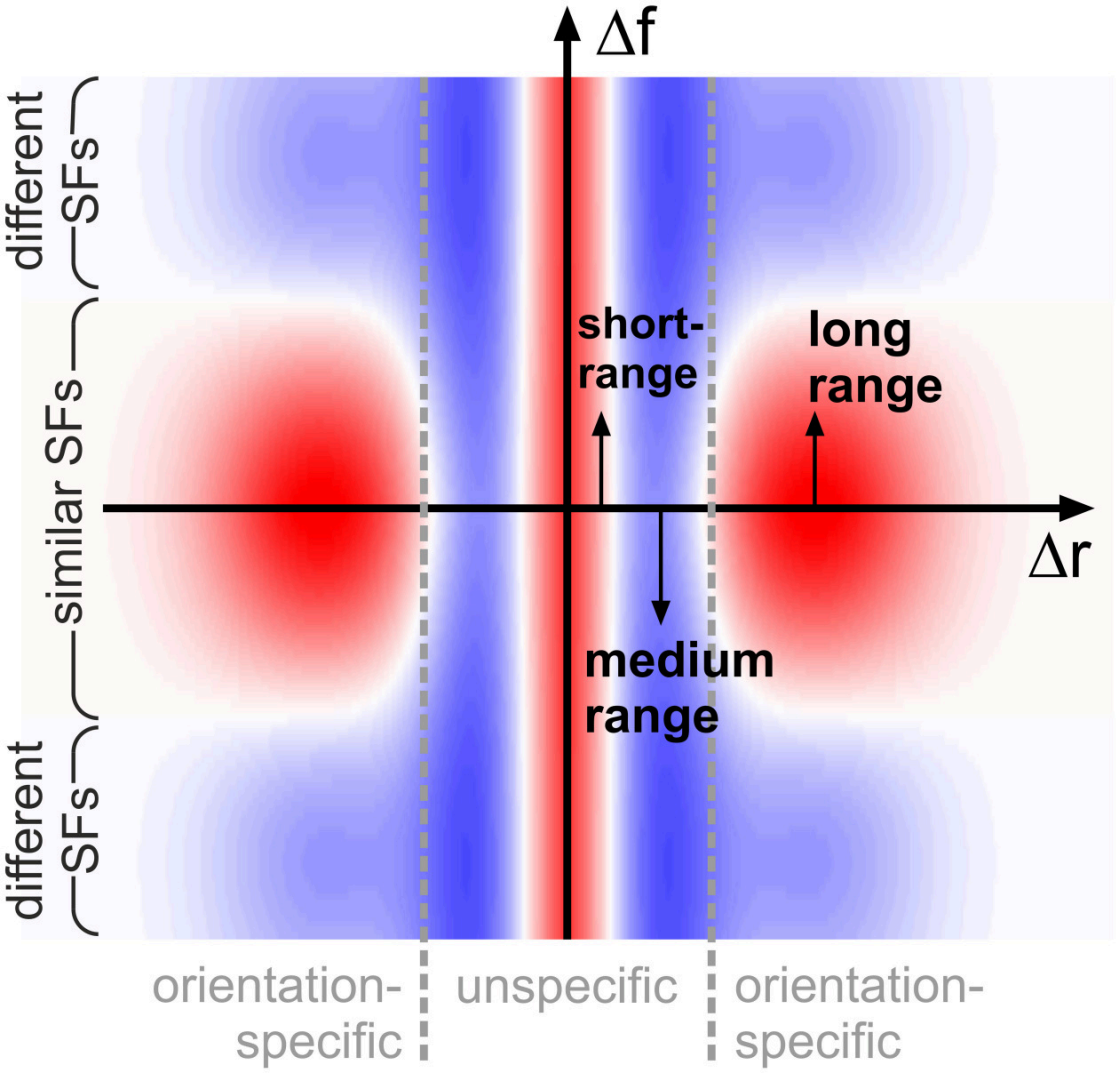
**Schematic representation of interactions**. Coupling scheme implied by our findings, shown for feature detectors with similar orientation preferences: antagonistic interactions in space (on small and intermediate distances Δ*r*, horizontal axis) are complemented by antagonistic interactions in spatial frequency (vertical axis, Δ*f*) for long spatial distances. Excitatory and inhibitory interactions are shown in red and blue shading, respectively. For clarity of illustration, we do not show that interaction length scales in addition depend on spatial frequency.

A single, common functional principle emerges when putting these observations into context with our knowledge about neural activation and detection thresholds if grating patches are smaller than 1°: typically, neural activation increases and detection thresholds decrease when the diameter of the patches becomes larger (Kretzberg and Ernst, 2013). This suggests an interaction profile resembling a Mexican hat: short-range excitation combined with medium-range inhibition. Now considering the orientation-specific interactions, a second Mexican-hat profile emerges in spatial frequency space: excitation if frequencies are close, and inhibition if frequencies are further apart. Functionally, Mexican-hat interactions are closely related to edge detection and image compression (e.g., see examples in Ernst, 2013): stimuli consisting of similar features are suppressed (low neural activity, Sillito et al., 1995; Levitt and Lund, 1997), while stimuli consisting of dissimilar features are enhanced (high neural activity, Sillito et al., 1995; Levitt and Lund, 1997). More complex interaction patterns (surrounds) which might be used by the brain to detect specifc patch configurations have also been reported from physiological studies (Walker et al., 1999). In our situation, the Mexican-hat in Cartesian space will suppress configurations with multiple, closely spaced patches (of any orientation and spatial frequency) in favor of configurations with more widely spaced, or isolated, patches. At the same time, the Mexican-hat in spatial frequency space will suppress configurations containing many spatial frequencies, while enhancing configurations with a single spatial frequency—provided that the patches have similar orientations, since this latter interaction is orientation (difference)-specific (Figure 9).

The finding of suppression in configurations with multiple, closely spaced patches resembles crowding phenomena, i.e. general detrimental effects of nearby probe stimuli on the perception of test stimulus attributes. Crowding effects were first observed in letter identification, but appear in a large variety of tasks (see Levi, 2008, for a comprehensive review). However, crowding effects mostly concern object feature identification and discrimination, but hardly object detection (Pelli et al., 2004). Particularly, crowding does *not* affect the apparent contrast of the test stimulus (Levi, 2008). Further, crowding effects are quite feature specific (Herzog et al., 2015). On the contrary, the inhibitory effects of close grating patch spacing reported here are effects on *contrast detection*, and were observed irrespective of orientation alignment and spatial frequency homogeneity. We therefore conclude that the inhibitory interaction for close spacings are better understood as lateral or surround masking effects (see Levi, 2008, for further aspects of distinguishing crowding from masking phenomena). Masking effects of surround gratings on central grating patches were extensively studied by Xing and Heeger (2000, 2001). Center (test) and surround (inducer) gratings were separated by a thin annulus, and the test contrast was matched to a reference grating of equal size, but without surrounding stimulus. The stimulus geometry in Xing's and Heeger's experiments compares to ours in the short (1°) spacing, since there the patches were separated by just 0.41° space of background luminance. Results showed that the test grating had lower perceived contrast than the reference grating, even when inducer contrasts were low. This result was practically independent of the spatial frequency of the gratings, but orientation difference of center and surround diminished the inhibitory contextual influence. In a similar center-surround arrangement Bruchmann et al. (2010) studied the temporal dynamics of the center-surround interaction, and consistently found evidence for inhibitory effects of the surround masker. Consistent with these results, we conclude that, despite some orientation selectivity, net contextual influence is inhibitory in the near surround.

Orientation-specific interactions are well known from electrophysiological studies on contour integration and are already observed in the form of firing rate enhancements for configurations of only two aligned edges (Ito and Gilbert, 1999). These effects increase in strength with an increasing length of a contour embedded into a randomly oriented background (Li et al., 2006). Optical imaging confirms these findings, and allows us to observe modulatory effects in a spatially extended manner (Gilad et al., 2013). Note that while our study locates all interactions within a single cortical layer, in the real brain different types of contextual interactions are typically located in different visual areas, as e.g., contour integration might be performed not in V1 but in V2 or in V4 (Chen et al., 2014). Moreover, psychophysical studies have shown that in contour integration, spatial frequency and orientation alignment information are combined to yield higher detection performances than expected from the additive combination of the single cues (Persike and Meinhardt, 2015a). Furthermore, if spatial frequencies in contour and background become homogeneous, detection performance is enhanced (Persike et al., 2009; Persike and Meinhardt, 2015b), similar to the effects observed with SF-homogeneous plaids in the 2° condition.

The functional role of the Mexican hat profile in spatial frequency is a potential advantage in reaching unique shape descriptions from single spatial scales. Studies on the detectability of simple global shapes have shown a detection advantage for shapes formed by parameter homogeneous Gabor patches, compared to heterogeneous Gabor elements (Saarinen et al., 1997; Saarinen and Levi, 2001). The advantage was found to be relatively independent of orientation alignment, and stressed the benefit of parameter homogeneity, in contrast to mixed configurations (Saarinen et al., 1997). These findings correspond to our finding of enhanced plaid detectability for orientation and spatial frequency homogeneous grating patches. However, evidence for suppressive interaction among different spatial scales is closely bound to contextual interactions in 2D configurations. Studying the interaction of different spatial scales at the same retinal location has revealed gradual decline of facilitation among grating patches when spatial scale difference is increased, until independence is reached for far apart local carrier frequencies of the grating stimuli (Watson, 1982). While the bandwidth of the psychophysical tuning function is close to the bandwidth of the grating patches, there is no evidence for inhibitory interactions among spatial frequency channels at one retinal location (see Meinhardt, 2001, p. 417). In their seminal psychophysical study on lateral grating patch interactions Polat and Sagi (1993) also studied contextual interactions for spatial scale differences of test and flankers. Results showed that the biphasic contextual response profile was attenuated for increasing spatial scale differences, but there was no change of the form of the profile, indicating no suppressive interactions for larger spatial scale differences. However, Polat and Sagi (1993) tested 1D contextual configurations of co-aligned stimuli, but not 2D configurations with collinear and collateral stimulus arrangements, as done here. Electrophysiological studies aiming at measuring a complete contextual interaction topography in 2D (Kapadia et al., 2000) have unfortunately not yet explored whether the contextual response field changes qualitatively if there is a spatial scale difference of central test and peripheral probe stimulus. More data are needed to settle the constraints for the relationship of object descriptions on different spatial scales and their neural underpinnings.

### 4.2. Similarities to natural image statistics

The observation that the statistics of plaid configurations in natural images is similar for a wide range of inter-patch distances is not surprising: many studies have shown (albeit sometimes w.r.t simpler features) that natural images have self-similar structures, i.e. that their statistics are invariant with respect to the particular observation scale (e.g., see Ruderman, 1997). For large inter-patch distances (in our case 2°), the visual system seems to realize interactions that enhance feature combinations with higher likelihoods to occur in “nature.” But why does this parallelism fail at 1°? Apart from the trivial explanation that there might be no reason at all, or that biophysical constraints prevent the brain from realizing the necessary neural couplings, there might be one functional explanation: Instead of just enhancing representation proportionally to their likelihood, our brain might rather be interested in enhancing representations *only* when they appear in contexts normally leading to reduced saliency. For example, in contour integration a continuous curve is much easier to follow and to integrate than a broken curve consisting of isolated, colinearly oriented line segments separated by “open” space: the larger the separation of elements, the less salient are contours (Mandon and Kreiter, 2005). Enhanced processing with larger distances would help to bridge the gaps and allow to detect the contour, in particular if these gaps would be filled with distractor elements. This example bears a close resemblance to a plaid consisting of four iso-oriented, frequency-homogeneous gratings, where we find suppression when the elements are very close (1°), but enhancement when the elements are well separated (2°). Here one might speculate that the visual system suppresses “trivial” while enhancing “surprising,” or more challenging feature conjunctions.

### 4.3. Model comparison and parameter discussion

The two variants of our network model are extremely simplified versions of more complex approaches (e.g., Li, 1998; Ichida et al., 2006; Hansen and Neumann, 2008). This reductionist approach was taken for two reasons. First, for revealing computational mechanisms as succinctly as possible, and second, for reducing free parameters as far as possible. Even so, model A is still susceptible to overfitting, as indicated by its match to the experiment being far better than expected from the confidence intervals of the experimental results. Model B is superior in the sense of avoiding overfitting: except from one configuration, still all experimental results are explained within their confidence intervals.

Short-range excitatory couplings, which would have been implemented as self-interactions between orientation columns, were not included in our model. Mathematically, *including* these interactions is equivalent with re-scaling the feedforward and recurrent input strength *without* having such interactions. Consistent with previous results, our model also requires interaction ranges to depend on SF preference of the feature detectors involved. It turned out that this interaction has to scale less than exactly anti-proportionally to SF (Polat and Sagi, 1993). Furthermore, we also tested whether cross-SF interactions are required to be orientation-specific. Without this specificity, the match between experimental results and model predictions was far worse. A comparison of our interaction scheme to association fields obtained from studies on contour integration is, unfortunately, not possible. Since our visual stimuli sample orientation (difference) space only extremely sparsely, it is difficult to predict interactions for orientation preferences not being parallel or orthogonal to each other. In addition, we obtain the best fit of the model by *not* having different interaction strengths between parallel and aligned configurations with same orientation. This feature is in contrast to interactions predicted from contour integration studies where parallel configurations (“ladders”) are much harder to perceive than aligned configurations (“snakes”) (Bex et al., 2001; May and Hess, 2007; Vancleef and Wagemans, 2013).

### 4.4. Outlook

Can our approach make predictions for even more complex plaids? Our interpretation of the interactions as one functional principle (Mexican hat) extending over multiple feature dimensions makes possible some “educated guesses.” For example, if a 2° configuration is “filled” by adding five patches in the spaces between the original plaid, we expect inhibition to kick in and raise detection thresholds. This would be consistent with our functional explanation that a closely spaced, 3 × 3 configuration of identical patches would be not surprising at all, but considered as a homogeneous texture possibly just being the background of much more interesting image features. It would also be interesting to restrict the image analysis to “informative” patch configurations, as e.g., has been done for oriented edge statistics by requiring human observers to label contours belonging to the same object (Geisler and Perry, 2009).

## Author contributions

All authors contributed equally to the conceptualization of the study. GM set up the basic design, performed the data analysis and contributed the interpretation. MP conducted the experiments and data preparation. UE set up the models and image analysis, performed the simulations, and analyzed model and image analysis results. AS performed the initial simulations for model A. All authors were involved in writing, preparation of the manuscript and final approval. All authors agree to be accountable for all aspects of the work in ensuring that questions related to the accuracy or integrity of any part of the work are investigated and resolved appropriately.

## Funding

This work was supported by the BMBF (Bernstein Award UE, grant no. 01GQ1106) by the Volkswagen Foundation (SmartStart grant to AS), and the DFG (Priority Program 1665, grant ER 324/3).

### Conflict of interest statement

The authors declare that the research was conducted in the absence of any commercial or financial relationships that could be construed as a potential conflict of interest.
