# Supplementary material for: Contextual Interactions in Grating Plaid Configurations Are Explained by Natural Image Statistics and Neural Modeling
Source: Front Syst Neurosci. 2016 Oct 4;10:78. doi: 10.3389/fnsys.2016.00078 (PMC5048088; doi:10.3389/fnsys.2016.00078)
Supplement: Supplementary file 1 [file DataSheet1.PDF]

Contextual interactions in grating plaid configurations are explained by natural image statistics and neural modeling

Supplemental Information

U. Ernst, A. Schiffer, M. Persike and G. Meinhardt

1 Experiments

Estimation of threshold reduction due to spatial probability summation. For computing an expected threshold contrast for a given plaid configuration, we consider a situation where k stimuli are processed independently and in parallel. Assuming the detection event happens if at least one of the k stimuli is detected, less contrast is necessary compared to a single stimulus. The nonlinear summation effect due to an increasing number of independent stimulus events is referred to as *probability summation* (Robson and Graham, 1981; Watson, 1982). If detection is by probability summation among k stimuli, the probability of detection for a given contrast value Θ is

$$P(\Theta) = 1 - \prod_{j=1}^{k}(1 - P_j(\Theta)). \tag{1}$$

If the k stimuli are equally detectable with probability P_s, equation (1) simplifies to

$$P(\Theta) = 1 - (1 - P_s(\Theta))^k . \tag{2}$$

Assuming the Weibull model for the psychometric function (Quick, 1974; Green and Luce, 1975)

$$P(\Theta) = 1 - \exp\left(-(\Theta \|\mathbf{R}\|)^\beta\right), \tag{3}$$

with $\|\mathbf{R}\|$ being the Minkowski norm of neural subunit responses to the stimulus, and β the shape parameter controlling steepness, the psychometric function (2) for probability summation reads

$$P(\Theta) = 1 - \exp\left(-k(\Theta \|\mathbf{R}\|)^\beta\right). \tag{4}$$

[1] It is convenient to consider normalized contrasts $\hat{\Theta} = \Theta/\Theta_0$, where Θ_0 is the threshold contrast assigned to the constant probability of detection $P_0 = 1 - 1/e$ in equation (3) , i.e. P_0 is the probability of detection that corresponds to the threshold condition $\Theta_0\|\mathbf{R}\| = 1$. On normalized contrasts the psychometric function for probability summation among k equally detectable stimuli becomes

$$P\left(\hat{\Theta}\right) = 1 - \exp\left(-k\hat{\Theta}^\beta\right). \tag{5}$$

[1] It is a major advantage of Quick's approach, which combines the Weibull model for the psychometric function and the Minkowski norm for describing nonlinear pooling of neural responses that the contrast parameter is separable from the neural subunit responses despite the nonlinear effects of probability summation. The net subunit responses are conceived as resulting from a convolution of the receptive fields' weighting functions with the luminance profile of the stimulus. This approached is used in many models of contrast detection (e.g. Wilson and Bergen (1979); for a comprehensive overview see du Buf (1992)).

Considering equation (5) at P_0 is used to derive the normalized threshold contrast for probability summation among k equally detectable stimuli. This resolves to

$$\hat{\Theta}_0 = k^{-1/\beta}. \quad (6)$$

Equation (6) shows that the shape parameter β rules the degree of threshold reduction due to probability summation. Measuring contrast thresholds with the adapted version of the method of limits used here for various grating patterns and fitting Weibull functions to the normalized contrast data showed that estimates of β were in the range [3.5, 6] (Meinhardt, 1999). The method of constant stimuli in a 2 alternative forced choice task leads to shallower slopes of the psychometric curve, and estimates of β are found in the range [2.7, 4.6] (Robson and Graham, 1981). We decided to use the range [3.5, 4] for the estimated threshold contrast reduction due to probability summation. This range is a conservative estimate but it takes into account that the methods of limits with averaging the results from 2 up-down runs leads to a smaller variance of threshold contrasts than forced-choice methods (for details, see discussion in (Meinhardt, 1999, 2000). Inserting in equation (6) leads to estimates of $\hat{\Theta}_0 = 0.673$ for $\beta = 3.5$ and of $\hat{\Theta}_0 = 0.707$ for $\beta = 4$. This means that we expect threshold reduction in the range of about 29% to 33% due to probability summation when a single grating patch is presented at 4 spatial positions simultaneously.

2 Cortex model

Numerical implementation and parameter adaptation In the beginning of each model simulation, the network was always initialized with zero activity in all units, and its dynamics computed using an Euler scheme with integration step size $\Delta t \ll \tau := 1$ until convergence to a steady–state solution. Our weight adaptation schemes (see below) assured that coupling was never so strong as to induce a divergent activation exponentially growing over time. The external input value of $J^{\text{ffw}} = 2$ was chosen such that without recurrent input, and with a minimum threshold of $J^{\text{thr}} = 1$ (cf. parameter initialization in **Table 1**), the resulting (dimensionless) activation A is 1.

Adaptation of model parameters was performed by a stochastic gradient descent on the free parameters $\vec{q}$ of each model. Starting from an initial parameter set $\vec{q_0}$, a change was applied to a random parameter q_i and accepted if the mean quadratic distance between the predicted and measured detection thresholds $\overline{\Theta}_c$ and $\hat{\Theta}_c$ decreased. If this was not the case, the parameter change was discarded. This procedure was repeated until a (local) minimum in parameter space was reached. The initial values for the parameters were selected from a uniform distribution between a minimum and maximum initial value, and parameter adaptation was bounded (clamped) by a minimum and maximum possible value (see **Table 1**). For each model, we started from 500 initial conditions. As target thresholds, we used the 9 average thresholds for each plaid category for each patch distance shown in **Figure 5B (main manuscript)**, and we additionally required that the threshold predicted from running the simulation *without* recurrent interactions matches the average threshold predicted from probability summation of the single patch thresholds (center of gray bars in **Figure 5B (main manuscript)**).

In model A, using "assumption-free" interactions implies $N^2 - N = 240$ degrees-of-freedom (df). This number was greatly reduced by taking symmetry constraints into account, namely that (a) $W = W^T$, that (b) connections are invariant with respect to shifts in space, and that (c) connections between a pair of plaids are invariant with respect to a rotation of that pair configuration in space. Satisfying these symmetries reduces the df to 15. Since 1° and 2° plaids imply modeling interactions on two different spatial scales, interactions in this first approach are defined by two coupling matrices $W^{[1^\circ]}$ and $W^{[2^\circ]}$, respectively, with 15 free parameters $\vec{w}^{[1^\circ]}$ and $\vec{w}^{[2^\circ]}$ each. We adapted these parameters simultaneously for 1° and 2° distances, with the full parameter set consisting of 32 components $\vec{q} := \{\vec{w}^{[1^\circ]}, \vec{w}^{([2^\circ]}, A^{\text{max}}, \kappa\}$.

For model B, parameters were also adapted simultaneously for 1° and 2° distances, with the parameter set consisting of 11 components $\vec{q} := \{G^{\text{iso}}, \mu^{\text{iso}}, \sigma^{\text{iso}}, G^{\text{ori}}, \mu^{\text{ori}}, \sigma^{\text{ori}}, G^{\text{frq}}, \mu^{\text{frq}}, \sigma^{\text{frq}}, A^{\text{max}}, \kappa\}$. These parameters quantify interaction strengths

w_{ik} via Gaussian functions

$$G(G_0, \mu, \sigma, d_{ik}) = G_0 \exp\left(-\frac{(d_{ik} - \mu)^2}{2\sigma^2}\right) \quad (7)$$

defined over the distance d_{ik} between two receptive fields (i.e., the distance between patches i and k in a plaid):

$$w_{ik}^{\text{iso}} = G(G^{\text{iso}}, \mu^{\text{iso}}, \sigma^{\text{iso}}, d_{ik}) \quad (8)$$

$$w_{ik}^{\text{ori}} = G(G^{\text{ori}}, \mu^{\text{ori}}, \sigma^{\text{ori}}, d_{ik})\delta_{\Phi_i,\Phi_k}\delta_{f_i,f_k} \quad (9)$$

$$w_{ik}^{\text{frq}} = G(G^{\text{frq}}, \mu^{\text{frq}}, \sigma^{\text{frq}}, d_{ik})\delta_{\Phi_i,\Phi_k}(1 - \delta_{f_i,f_k}) \quad (10)$$

Here, Kronecker's δ was used to implement specificity of interactions as described above. The total interaction strength w_{ik} between units i and k is then obtained by adding these three contributions, $w_{ik} = -w_{ik}^{\text{iso}} + w_{ik}^{\text{ori}} - w_{ik}^{\text{frq}}$. As an additional assumption, we include that the range of interactions (a) and (b) scales with spatial frequency (Polat and Sagi, 1994), i.e. $\mu \propto f^{\nu}$ and $\sigma \propto f^{\nu}$. $\nu = 1$ realizes a linear scaling, while $\nu < 1$ or $\nu > 1$ realize a supra-linear or super-linear scaling, respectively. Interactions w^{frq} *between* different spatial frequencies f_i and f_k were scaled to the average of the ranges for the single frequencies. After fixing ν at its "optimal" value of $\nu = 0.5$, found through extensive simulations (not shown).

Table 1. Initialization and constraints of parameter adaptation. Parameters were initialized with values drawn from a uniform distribution between **init (min)** and **init (max)**. During adaptation, clamping was used to ensure that no parameter went below **adapt (min)** or above **adapt (max)**. For adaptation, the **add**-rule changed a parameter q according to $q^{\text{new}} = q^{\text{old}} + \epsilon\eta$, and the **mul**-rule changed a parameter q according to $q^{\text{new}} = q^{\text{old}}(1 + \epsilon\eta)$. ϵ was chosen to be $\epsilon := 0.02$, and η was a random number drawn from a uniform distribution between -1 and $+1$. Dashes indicate parameters that were initialized randomly but not adapted. The last row shows average and variation of the final values for the best models after adaptation

model	parameter	init (min)	init (max)	adapt (min)	adapt (max)	rule	average final value
A	w (2 x 15)	-0.05	0.05	-10	10	add	see **Figure 6 (main ms.)**
B	G^{iso}	0.1	0.1	0	10	mul	0.57 ± 0.09
B	G^{ori}	0.1	0.1	0	10	mul	0.43 ± 0.03
B	G^{frq}	0.1	0.1	0	10	mul	0.95 ± 0.29
B	μ^{iso}	0.5	3	0	10	mul	2.38 ± 0.01
B	μ^{ori}	0.5	3	0	10	mul	4.48 ± 0.16
B	μ^{frq}	0.5	3	0	10	mul	4.89 ± 1.80
B	σ^{iso}	0.5	3	0	50	mul	0.33 ± 0.01
B	σ^{ori}	0.5	3	0	50	mul	1.49 ± 0.05
B	σ^{frq}	0.5	3	0	50	mul	2.83 ± 1.60
A,B	J^{thr}	1	2	–	–	–	1.51 ± 0.29, 1.50 ± 0.31
A,B	A^{max}	1	5	0	∞	mul	1.26 ± 0.84, 1.09 ± 0.74
A,B	μ	1	2	0.1	10	mul	1.99 ± 0.85, 1.11 ± 0.15

3 Natural image statistics

This subsection provides detailed information about natural image analysis, namely for the whitening procedure and for computing the likelihoods for the presence/absence of oriented features (gratings) based on a generative model.

Whitening. The whitening filter was constructed from the average amplitude spectra of each image ensemble $\mathcal{E}$. More specifically, we applied a 2D–Fourier transform to each image converted to gray scale, and computed the absolute value of the Fourier coefficients. Hereby we focused only on the coefficients for the wave vector orientations 45° and -45°, thus matching the orientations used in the psychophysical experiment. The corresponding spectra range from frequencies of about 0.001-0.01 periods/pixel up to the Nyquist frequency of 0.5 periods/pixel. The coefficients were averaged over the two orientations and all images in a set, yielding a whitening function $w(k)$ that depends on wave number k. Within the analyzed frequency range, $w(k)$ is close to a power law with its exponent between 1.1 and 1.2. The whitening filter in Fourier space $\mathbf{k}$ is then defined as $F_w(\mathbf{k}) := 1/w(k)$ with $k = |\mathbf{k}|$, thus effectively normalizing the amplitude spectrum over the corresponding image ensemble.

Patch and Plaid likelihoods. This subsection explains how $L(\mathbf{C}|\mathcal{E})$ and $L(\mathbf{c}|\mathcal{E})$ in

$$\Lambda(\mathbf{C}) \quad := \quad \frac{L(\mathbf{C}|\mathcal{E})}{\prod_{i=1}^{4} L(\mathbf{c}_i|\mathcal{E})} \tag{11}$$

were computed. Different plaid configurations $\mathbf{C} := \{\mathbf{c}_1, \mathbf{c}_2, \mathbf{c}_3, \mathbf{c}_4\}$ are described by their single components (patches) $\mathbf{c}_i := \{\varphi_{k_i}, f_{l_i}\}$, subsuming orientation φ_{k_i} and spatial frequency f_{l_i} of grating patch i. i counts the elements of a plaid in counter-clockwise direction, starting from the topmost patch (**Figure 1 (main manuscript)**). Orientation and spatial frequency take discrete values $\varphi_1 := \pi/2$, $\varphi_2 := -\pi/2$, $f_1 := f_{\text{low}}$ and $f_2 := f_{\text{high}}$.
To compute the likelihood $L(\mathbf{C}|\mathcal{E})$, we quantify to which particular plaid configuration $\hat{\mathbf{C}}$ a particular image region is most similar, and count the number of matches for each possible plaid $\mathbf{C}$ (four positions × two orientations × two spatial frequencies yield 256 different plaids, including all symmetric configurations) over the whole image ensemble $\mathcal{E}$. Since each plaid consists of four grating patches, this inference problem reduces to computing which particular grating patch $G(\varphi_{\hat{k}}, f_{\hat{l}})$ is most similar to a particular image patch $J(\mathbf{r})$, for all four patches in a plaid. Here, $J(\mathbf{r})$ represents a part of an image from ensemble $\mathcal{E}$ which has the same spatial extension as a grating patch G, with $\mathbf{r}$ denoting the position vector relative to its center. We obtained the orientation-spatial frequency index pair $\{\hat{k}, \hat{l}\}$ best explaining patch J by evaluating the expression

$$\{\hat{k}, \hat{l}\} \quad = \quad \text{argmax}_{k,l} \left\{ \log P\left(J | G_{\varphi_k, f_l}\right) \right\} \,, \tag{12}$$

where P is the conditional probability of observing image patch J given the presence of a grating patch G_{φ_k, f_l}, and obtained $L(\mathbf{C}|\mathcal{E})$ by counting matches for all possible image regions z via

$$L(\mathbf{C}|\mathcal{E}) \quad = \quad \sum_{z \in \mathcal{E}} \prod_{i=1}^{4} \delta_{\hat{k}_i^z, k_i} \delta_{\hat{l}_i^z, l_i} \,, \tag{13}$$

where δ denotes Kronecker's delta. The four image patches $J_i(\mathbf{r})$ were always taken from the same image region, and extracted around the center positions of the four patches in a typical plaid configuration. The sum over z runs over different images as well as over different regions sampled systematically from the same image. Correspondingly, the likelihoods for single patches are computed via

$$L(\mathbf{c}|\mathcal{E}) \quad = \quad \sum_{z \in \mathcal{E}} \delta_{\hat{k}^z, k} \delta_{\hat{l}^z, l} \,. \tag{14}$$

Generative model. For computing $P(J|G)$ in equation (12), one needs to specify a generative model for the occurrence of gratings in image patches. Here we assume that image patches $J(\mathbf{r})$ are stochastic realizations of Gabor functions $g_{\mathbf{p}}(\mathbf{r})$ with a modulation amplitude (contrast) A. $\mathbf{p} := \{\varphi, f, \sigma, \Phi\}$ are parameters of the Gabor, where φ is its orientation, f its spatial frequency, σ its half-width and Φ its phase:

$$g_{\mathbf{p}}(\mathbf{r}) \quad = \quad n_{\mathbf{p}}(\mathbf{r}) \frac{1}{2\pi\sigma^2} \exp\left(-\frac{\mathbf{r}^2}{2\sigma^2}\right) \tag{15}$$

$n_{\mathbf{p}}$ comprises a modulation term and a normalization term,

$$n_{\mathbf{p}}(\mathbf{r}) \quad = \quad \cos\left(\Phi + 2\pi f \left(\mathbf{r} \cdot \mathbf{e}_{\varphi+\pi/2}\right)\right) - \cos\Phi \exp\left(-2\left(\pi\sigma f\right)^2\right) , \tag{16}$$

and $\mathbf{e}_{\varphi+\pi/2}$ denotes the unit vector pointing in the direction perpendicular to the Gabor's orientation φ. Although Gabors are not exactly identical to the grating patches used in the experiment, they are much better suited for a computational analysis since their envelope is smooth and thus reduces boundary effects in filter operations.
If we assume a Gaussian statistic for the observation noise, it follows that

$$L_{\mathbf{p}} := \log P(J|g_{\mathbf{p}}) \quad = \quad -c \int_J \left(J(\mathbf{r}) - A g_{\mathbf{p}}(\mathbf{r})\right)^2 d\mathbf{r} \tag{17}$$

with a positive constant $c > 0$. Although images are discretized into pixel representations, here we use a continuous representation of space $\mathbf{r}$ which allows us to employ integrals instead of sums hence facilitating analytical evaluation of the corresponding equations. By evaluating the r.h.s. of equation (17), one obtains an expression with three terms,

$$L_{\mathbf{p}} \quad = \quad -c\left[\int_J J^2(\mathbf{r}) d\mathbf{r} + A^2 \int_J g_{\mathbf{p}}^2(\mathbf{r}) d\mathbf{r} - 2A \int_J J(\mathbf{r}) g_{\mathbf{p}}(\mathbf{r}) d\mathbf{r}\right] . \tag{18}$$

For a given image patch J, the first term is constant. For $\sigma > 1/(2f)$, the second term is also approximately constant, with its value being close to $A^2/(8\pi\sigma^2)$ with a remaining error below 0.25 percent. Hence for maximizing $L_{\mathbf{p}}$ it suffices to maximize the overlap $O_{\mathbf{p}}$ between Gabor and image patch given by $O_{\mathbf{p}} := \int_J J(\mathbf{r}) g_{\mathbf{p}}(\mathbf{r}) d\mathbf{r} = \langle J \star g_{\mathbf{p}} \rangle$. Determining the combination of orientation and spatial frequency best matching J *independently* of Gabor phase requires finding $\max_\Phi O_{\mathbf{p}}$, which is normally done by using a quadrature pair of Gabor filters in the following expression,

$$\max_\Phi O_{\mathbf{p}} \quad = \quad \sqrt{O_{\mathbf{p},\Phi=0}^2 + O_{\mathbf{p},\Phi=\pi/2}^2} . \tag{19}$$

Finally we rewrite equation (12), thus obtaining the desired maximum-likelihood estimator

$$\{\hat{k}, \hat{l}\} \quad = \quad \text{argmax}_{k,l} \log P\left(J|G_{\varphi_k, f_l}\right) = \text{argmax}_{k,l} \left\{\max_\Phi O_{\mathbf{p}}\right\} . \tag{20}$$

The Gabors' spatial extension σ does not need to be treated as a free parameter, since it has a fixed value determined by the geometry of the stimuli used in the experiment.
For reducing noise contributed by image regions with non-oriented, homogeneous content, we discarded image patches for which $\log P$ in equation (20) was below a threshold of 5% of the maximum of $\log P$ over all possible patches in a particular image.

References

du Buf, J. M. H. (1992). Modeling spatial vision at the threshold level. *Spatial Vision* 6, 25–60

Green, D. M. and Luce, R. D. (1975). Parallel psychometric functions from a set of independent detectors. *Psychological Review* 82, 483–486

Meinhardt, G. (1999). Evidence for different nonlinear summation schemes for lines and gratings at threshold. *Biological Cybernetics* 81, 263–277

Meinhardt, G. (2000). Detection of compound spatial patterns: further evidence for different channel interactions. *Biological Cybernetics* 82, 269–282

Polat, U. and Sagi, D. (1994). The architecture of perceptual spatial interactions. *Vision Res* 34, 73–78

Quick, R. F. (1974). A vector - magnitude model for contrast detection. *Kybernetik* 16, 65–67

Robson, J. G. and Graham, N. (1981). Probability summation and regional variation in contrast sensitivity across the visual field. *Vision Research* 21, 409–418

Watson, A. B. (1982). Summation of grating patches indicates many types of detector at one retinal location. *Vision Research* 22, 17–25

Wilson, H. R. and Bergen, J. R. (1979). A four mechanism model of threshold spatial vision. *Vision Research* 19, 515–522
